# Prevention of acid rock drainage formation through pyrite inhibition by silica coating

**DOI:** 10.1007/s11356-025-36131-x

**Published:** 2025-02-27

**Authors:** Dantie Claudia Butar Butar, Lena Alakangas, Hanna Kaasalainen, Erik Ronne

**Affiliations:** 1 Applied Geochemistry, Swedish School of Mines, Department of Civil, Environmental and Natural Resources Engineering, Luleå University of Technology, SE-971 87, Luleå, Sweden; 2 Geological Survey of Finland, Vuorimiehentie 5, 02150 Espoo, Finland; 3 Boliden AB, SE-101 20, Stockholm, Sweden

**Keywords:** Acid rock drainage, Pyrite, Sulfur, Waste rock, Leaching, Silica, Coating, Inhibition

## Abstract

Passive treatment of acid rock drainage (ARD) is a sustainable approach to control ARD, with sulfide inhibition by silica being a promising alternative. In a small-scale column leaching, a total of four cells loaded with pyritic waste rock (11 wt% S) from an operating Cu mine in Sweden were kept in a climatic chamber at a controlled temperature and humidity. The waste rock was leached for 11 weeks before treatment using alkaline silicate solution was applied, without pH buffer and adjuster. One cell was left untreated, whereas the others were treated with silicate solution as a source of dissolved silica, with and without H_2_O_2_ pre-oxidation. The pH in silica-treated cells generated leachate with circumneutral pH until the end of the leaching cycle, whereas sulfide oxidation accelerated in the absence of treatment. Leachate quality in all Si-treated cells improved, as evidenced by the suppressed release of sulfur and other metals (e.g., Al, Fe, Cu, Co, Mn, and Ni). Upon treatment with a longer contact time, silica (SiO_2_) layer developed on waste rock and inhibited pyrite. The layer remained stable upon extended exposure to air and water for up to 10 weeks after treatment. Despite forming a siliceous Fe–O phase, H_2_O_2_ pre-oxidation resulted in indirect oxidation of sulfides and other phases. With an excess of silicate solution and at alkaline pH, pyrite surfaces are devoid of coating and metal ions were mobilized. Finally, this study suggested that treatment of pyritic waste rock using silica can attenuate ARD formation and prevent metal leaching by pyrite inhibition and maintaining a circumneutral pH environment or both.

**Supplementary Information:**

The online version contains supplementary material available at 10.1007/s11356-025-36131-x.

## Introduction

The global demand for metals for economic and societal development, particularly clean technologies, has increased exponentially. In the last 10 years, Swedish ore production has nearly doubled the production of the previous decade. In Sweden, extraction of base and precious metals, e.g., Cu, Zn, Pb, and Ag, mainly originate from polymetallic sulfide-bearing ore deposits and may produce waste containing iron-sulfide minerals such as pyrite (FeS_2_) and pyrrhotite (Fe(_1-*x*)_S). In 2022, the annual generation of waste rock from Swedish non-ferrous mines was approximately 32.6 Mt (Liljenstolpe et al. 2023). Waste rock considered sub-economic (i.e., below the cut-off mineable grade) is transported and deposited in heaps, left under ambient conditions. Over time, waste rock exposed to oxygen and water enables sulfides to oxidize, and the weathering products can form acid rock drainage (ARD) unless prevented in time. In Sweden, waste rock containing low concentrations of sulfides can be utilized for construction at the mine (on-site). Waste rock containing high concentrations of sulfides might be considered for waste valorization to produce sulfuric acid (H_2_SO_4_). Waste rock with medium sulfide content falls into neither category, thus requiring treatment to prevent ARD formation.

ARD is one of the most critical environmental problems that cause acidification and metal(loid) contamination of water and soil (Peppas et al. 2000), as well as impairs waterways (Akcil and Koldas 2006). Furthermore, the discharge of ARD into receiving water bodies causes increased turbidity and sedimentation, acid–base imbalance (Acharya and Kharel 2020), and biotic impairment, which often occurs via immediate toxicity, habitat destruction by metal precipitates, or alterations of nutrient cycle. In turn, water impairment due to ARD remains unsuitable for domestic consumption, as well as agricultural and industrial uses (Skousen et al. 2017). ARD is primarily characterized by low pH, high total dissolved solids, and dissolved concentrations of metal(loid)s. As the sulfides react with oxidants (O_2_ and Fe^3+^) and water, elements that are associated and hosted within them will also be released simultaneously and further render the discharge high in dissolved metal(loid)s, such as As, Co, Ni, Pb, and Zn. The acidic environment may dissolve silicate minerals and mobilize additional elements, such as Al. If the net neutralizing capacity in the waste rocks is low, presumably due to the lack of readily soluble pH buffering minerals (i.e., calcite/CaCO_3_), a measure should be taken to treat the water or prevent the formation of ARD, as ARD is hard to suppress once it initiates.

During the mining operations, the most common method to limit ARD is active water treatment, i.e., treating by active addition of lime (CaO), hydrated lime (Ca(OH)_2_) anhydrous ammonia (NH_3_), or NaOH to neutralize acidity (Skousen et al. 1998). However, this method is not cost- and resource-efficient as it requires continuous consumption of chemical reagents, ongoing expense for operation and maintenance (Skousen et al. 2017), and considerable manpower for continuous input for alkalinity to neutralize ARD (Tu et al. 2022). In the long term, active treatment is also associated with increased maintenance and management cost (Chen et al. 2021), costly investment for membrane separation process (Aguiar et al. 2016) and handling of sludge (Tu et al. 2022) as secondary pollutant, thus is not sustainable. In water treatment, where ARD has already developed, the method aims to mitigate the effect of spreading contaminated water. Accordingly, the current best practice is at-source prevention of ARD rather than treating the water. ARD prevention methods include, for example, physical barriers, inhibition of reactive surfaces by bacteria, and chemical passivation (Sahoo et al. 2013). Physical barrier, such as mine waste cover (dry or wet) over the waste (Hallberg et al. 2005; Jia et al. 2015), effectively retards sulfide oxidation and is typically applied as a measure at mine closure. However, it is a site-specific method which relies on on-site climate, hydrology, and reactivity of the mine waste (Mine Environment Neutral Drainage Program (MEND) 2004). For instance, desulphurized tailings as cover to prevent ARD have been proven suitable in a humid climate (Demers et al. 2008).

Chemical passivation, alternatively known as inhibition of pyrite surfaces, effectively reduces ARD at its source (Tu et al. 2022) and is a cost-effective method to prevent pyrite oxidation (Park et al. 2019). Inhibition is analogous to the principle of oxygen barrier coverage whereby a passivating layer attaches to the mineral surface to form a dense coating to inhibit surface oxidation, surface dissolution, or surface adsorption (Li et al. 2024) and eventually block acid production (Zhang et al. 2023). Inhibition may complement covers since it has proven effective in suppressing pyrite oxidation by reducing their reactivity. Previous inhibition research involved the use of secondary raw materials (SRM), for instance, green liquid dregs (Alakangas et al. 2013), lime kiln dust (Nyström et al. 2019a), cement kiln dust (Nyström et al. 2019b), blast furnace slag (Nyström et al. 2019b), and fly ash (Alakangas et al. 2013; Nyström et al. 2019b; Pérez-López et al. 2007, 2009), to neutralize pH and promote the formation of hydrous ferric oxide (HFO) which can prevent pyrite from reacting with oxidants. The amount of SRM added to the waste rock is limited to 5 wt% for the treatment to become economically feasible if transport cost is factored in (Alakangas et al. 2013, 2014).

Possible risks and concerns are raised regarding inhibition types and methods as they may be attributed to specific issues, e.g., eutrophication potential in downstream water bodies by phosphate-based coatings (Evangelou 2001; Kollias et al. 2019), or the toxicity and degradability of organic coatings by microorganisms (Jiang et al. 2000), and organosilane encapsulation methods (Dong et al. 2020, 2022) which are known to be less selective, unavailable in nature and hence expensive (Ouyang et al. 2015). Recent research in pyrite inhibition includes, for instance, PropS-SH/halloysite nanotube (HNT)-Benzotriazole (BTA) (PSHB) (Li et al. 2021) and PropS-SH-tannic acid coatings (Li et al. 2023). However, these methods were applied to pure pyrite samples, hence their applicability to complex, heterogeneous pyritic samples using sources available in nature as well as their geochemical implication still require further investigation.

Concerns regarding applicability, cost, and environmental feasibility raise the need to develop an inert and environmentally friendly coating from readily available natural sources. Passivation coatings by silica and silicate are more environmentally friendly (Zhang et al. 2023). In addition, silica is omnipresent in geological materials, e.g., soil, sand, suspended colloidal clays and related minerals (Iler 1979) and biogenic sources, as well as in many siliceous industrial remnants, e.g., slag and tailings. Due to its environmentally benign nature, the suitability of using silica for pyrite inhibition has garnered attention. Silica application to inhibit pyrite oxidation has been investigated previously, marked by the formation of a silicate-stabilized coating on the pyrite surface (Fan et al. 2017; Zhang and Evangelou 1998), Fe-silica passivating layer on pyrite (Kang et al. 2024; Kollias et al. 2022) that is even stable at a pH 2.5–4.0 (Evangelou 1996; Kargbo and Chatterjee 2005), and silica precipitate on pyrite at neutral pH (Bessho et al. 2011).

Despite their prevalence, comprehensive studies involving silica for pyrite inhibition primarily include a pre-oxidation step by the addition of H_2_O_2_ in a buffered solution (pH 4–6) for the generation of ferric ions (Fe^3+^) (Kang et al. 2024) and later to form a silicate-stabilized ferric oxyhydroxide at a circumneutral pH. Most research on silica application for pyrite inhibition has predominantly tested on pulverized monomineralic pyrite samples (Bessho et al. 2011; Dong et al. 2020, 2022; Evangelou 2001; Fan et al. 2017; Kargbo and Chatterjee 2005) or pre-treated samples to remove secondary phases (Dong et al. 2022; Kollias et al. 2022). Therefore, the stability and sustainability of passivation technology in a complex environment is unclear (Chen et al. 2021). Additionally, previous treatment has been mainly conducted by applying coating solutions with stirring (Dong et al. 2022; Wang et al. 2019), mixed with buffer solution, such as CH_3_COONa buffer (Kang et al. 2024; Kollias et al. 2022) or NH_4_OH (Dong et al. 2020) for pH adjustment in order to favor the precipitation of some mineral phases (Butler and Brase 2024), which eventually does not warrant ease and suitability for field application. The examination of formed coating and effectiveness of pyrite inhibition on heterogeneous materials, as well as the geochemical characteristics of the leachate also have received limited attention, which limits the use of silica coating technologies (Butler and Brase 2024). Furthermore, the effect of repeated addition and longer contact time of silica has not been well established. Thus, to fill the research gaps, this study aims to prevent ARD formation through pyrite inhibition in waste rock by using an alkaline silicate solution as the source of dissolved silica without pH buffer and adjustment. Analyses of the solid and liquid phases, comparative geochemical characteristics of the leachate of (un)treated waste rock, and implication of this study will be discussed.

## Materials and methods

### Waste rock characteristics and analyses

The Kristineberg Mine, owned by Boliden Mineral AB, is situated 120 km west-northwest of Skellefteå in Västerbotten county, Sweden. Since the production began in 1940 until today, a total of 33 Mt of ore have been mined with an average grade of 1.2 g/t Au, 38 g/t Ag, 1% Cu, and 3.8% Zn (Bjänndal and Bradley 2023). The Kristineberg Mine is an operating mine, hosting a polymetallic Volcanogenic Hosted Massive Sulfides (VHMS) deposit in the locality, which encompasses Rävliden, Rävliden North, Rävlidmyran, Hornträsksviken, and Kimheden mines (Bjänndal and Bradley 2023). Waste rock containing pyrite was collected from a heap in the Kristineberg area and the samples originated from the Kristineberg Mine. The rocks were relatively fresh to slightly oxidize upon collection and had been deposited in the heap for less than 1 year.

The rock sampling campaign was performed preferentially on waste rock boulders with visible sulfides and did not represent all the rock types in the heap. The expected sulfur content was approximately 10 wt%. Eight pyritic waste rock of boulder size were collected; some were selected and crushed into 4–6-mm fractions for the leaching experiment. Crushing, screening, and splitting of the waste rock for the preparation of the leaching experiment were carried out by ALS Piteå, Sweden, and the resulting particle sizes ranging from 4 to 6 mm were selected in this study for the leaching test. The remaining samples were sawed into 2-cm-thick rock slabs for µ-XRF (X-ray fluorescence) analysis, whereas another sample fraction was crushed and ground into powder for mineral characterization using XRPD (X-ray powder diffraction). Thin sections were prepared using the remaining sample for the SEM–EDS (scanning electron microscopy–electron dispersive spectrometer) and LA-ICP-MS (laser ablation-inductively coupled plasma-mass spectrometry) analyses.

For mineralogical analysis, rock powders were analyzed using a semi-quantitative X-ray powder diffraction (XRPD) (Malvern, PANanalytical Empyrean) at Luleå University of Technology. The rock powders were examined using Cu-Kα radiation at 30 kV and 20 mA conditions and a scan speed of 0.07°/s using a 20-mm mask. The scan time was set to 20 min, and the scan scope was 5–90°. Phase composition was identified by analysis of the diffraction patterns. Similarly, the formed Si precipitate was analyzed under the same conditions, except a 15-mm mask was utilized. The detection limit of XRPD with Rietveld refinement is < 1 wt% per phase. Both multi-phase identification and Rietveld refinement were performed using Profex software using BGMN database. To provide elemental distribution and relative correlation between elements, µ-XRF (Bruker M4 Tornado) at Luleå University of Technology for small spots (< 20-µm spot size) was done on rock slabs. SEM analysis (Zeiss Sigma 300 VP) was conducted equipped with an EDS detector to quantify the % mass or atom of the detected elements to analyze the morphology and identify the composition and elemental distribution of the solid phase. A high-resolution inductively coupled plasma–sector field mass spectrometry (ICP-SFMS) analysis was performed at ALS Scandinavia (Luleå) to measure the major and trace elements in the rock samples. To quantify the trace elements in pyrite directly on the rock samples, analysis using LA-ICP-MS was performed at Luleå University of Technology in addition to ICP-SFMS. LA-ICP-MS offers a wide range of applications for trace element quantification, including quantitative sub-ppm trace element analysis. In contrast to ICP-SFMS analysis of the whole rock, which quantifies the elemental concentration in the bulk solid samples, LA-ICP-MS targets quantifying trace elements present within pyrite. Thus, this study added LA-ICP-MS to ensure which elements can be used as suitable tracers or proxies for sulfide oxidation.

### Leaching experiment

#### Experimental setup

**Fig. 1:**
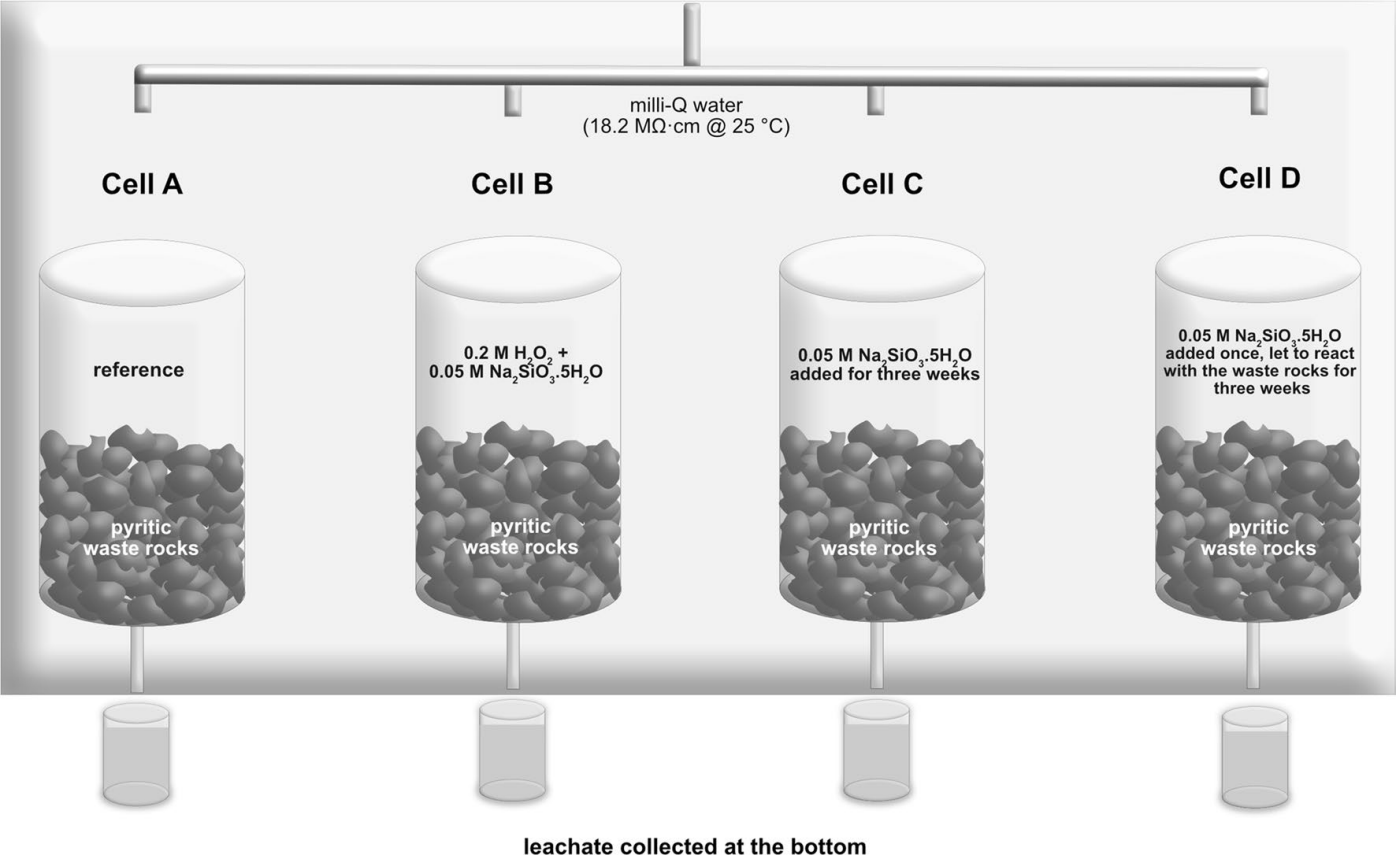
Experimental setup of all cells inside a climatic chamber, including a reference cell with waste rock only and three cells, where waste rock was subjected to Si treatment

Four leaching experiments on the waste rock were carried out to investigate pyrite inhibition. This study utilized four cells (Fig. 1) (500-mL polypropylene columns (⌀ 4 cm in diameter and 10-cm high)). Two hundred fifty grams of sulfidic waste rock with particle size between 4 and 6 mm was placed in each cell. At the base of the column, an inert cloth material was placed to prevent the loss of fine particles and hinder rock materials from clogging the outlet and tubes. The weekly liquid-to-solid ratio was kept constant at 1 mL of milli-Q water to 1 g of rocks. All cells were placed inside a climatic chamber with a temperature of 20 °C and humidity at 60%. Wet-dry cycles were involved in each leaching cycle. Before and after treatment, the waste rock was let to oxidize before flushing was conducted and leachate was collected on the last day of every cycle. A schematic view of the experimental set-up is shown in Fig. 1. Prior to the experiment, all equipment was pre-rinsed thoroughly in 5% HNO_3_ (ACS grade, Fisher Scientific). A blank sample of milli-Q water that percolated through an empty cell was submitted for analysis (Table 4 in the Appendix).

One of the four cells, cell A, i.e., untreated waste rock, was kept as a reference and let to oxidize with weekly flushing with Milli Q water. Three remaining cells were treated with an alkaline silicate solution at leaching cycle-11 (Table 1) after weeks of leaching using milli-Q water. A diluted sodium metasilicate pentahydrate (Na_2_SiO_3_·5H_2_O 0.05 M) solution (i.e., alkaline silicate solution) was used as the source of dissolved silica. The alkaline silicate solution was of reagent grade (≥ 95.0% purity from solid form, Sigma-Aldrich). The stock solution was prepared in a wide-mouth high-density polyethylene (HDPE) bottle to prevent possible leaching of Si from glass containers. The concentration of dissolved Si in the stock solution is 1.9 g/L based on the ICP-SFMS analysis (ALS Scandinavia, Sweden). In our experiment, no buffer solution was involved to alter the solution pH as opposed to other studies (Evangelou 2001; Fan et al. 2017; Kang et al. 2024; Kollias et al. 2022).

**Table 1:** Summary of the treatment in all cells

*Cell*	*Description*
*A*	Reference, untreated waste rock
*B*	Pyritic waste rock pre-oxidized with 100 mL 0.2 M H_2_O_2_ for 1 h, followed by addition of 150 mL Na_2_SiO_3_·5H_2_O 0.05 M. Leaching continued using milli-Q water throughout the rest of the experiment
*C*	Pyritic waste rock treated with 250 mL Na_2_SiO_3_·5H_2_O 0.05 M added every week for 3 weeks. Leaching continued using milli-Q water throughout the rest of the experiments
*D*	Pyritic waste rock treated with 250 mL Na_2_SiO_3_·5H_2_O 0.05 M once. The solution was let to react with the waste rock at a longer contact time (i.e., for 3 weeks) before leachate was drawn. Leaching continued using milli-Q water throughout the rest of the experiments

In cell B, a diluted 30% hydrogen peroxide (H_2_O_2_) (reagent grade, Merck) was added into the waste rock to accelerate the oxidation of sulfides before Si addition. H_2_O_2_ was let to react with sulfides for 60 min, and the reaction was evident by the formation of bubbles owing to the H_2_O_2_ dissociation in water and the formation of rust. H_2_O_2_ and Na_2_SiO_3_·5H_2_O solution were added slowly to ensure that the waste rock was flooded so that all pores were filled with the solution. In cell C, 250 mL of fresh silicate solution was added repeatedly for 3 weeks and let to react with the waste rock for 5 days in each leaching cycle. In cell D, 250 mL of silicate solution was added once, but the solution was left to react with the waste rock for 3 weeks before leachate was drawn. After treatment was completed, weekly leaching was continued in all cells using milli-Q water until the leaching was terminated at week 23. This corresponded to a total of 24 leaching cycles in cells A, B, and C and 22 leaching cycles for cell D, with the liquid/solid ratio equal to the number of leaching cycles.

### Leachate sampling and analyses

Leachate from all cells was sampled weekly, and the volume of the collected leachate was measured. Subsamples were collected and analyzed for solution pH, electroconductivity (EC), and redox condition (ORP) using a portable HACH multimeter (HQ2200), including pH (± 0.02), EC (± 0.5%), and ORP electrodes (0.05%). Prior to use, all electrodes were calibrated.

A subsample of the leachate was filtered through a 0.22-µm nitrocellulose (Merck Millipore) filter into polypropylene (PP) bottles, acid-washed in 5% HNO_3_ prior to use, and stored in darkness and cold (4 °C). Before analysis, the sample was acidified with 1 mL nitric acid (suprapur) per 100 mL at the ALS laboratory. Major and trace element concentration was analyzed using inductively coupled plasma atomic emission spectroscopy (ICP-AES) and inductively coupled plasma sector field mass spectrometry (ICP-SFMS) at SWEDAC-accredited ALS Scandinavia in Luleå, Sweden. The analysis was performed based on US EPA Method 200.7 (modified) and 200.8 (modified) or quantitative screening analysis for 70 elements. Anions were not determined, but the sulfur measured by ICP-AES is assumed to represent sulfur in the form of sulfate, confirmed by geochemical calculations.

Geochemical calculations, including aqueous species distribution and mineral saturation state, were performed using PHREEQC version 3.7.3 (Parkhurst and Appelo 1999) and the *WATEQ4F.dat* thermodynamic database (Ball and Nordstrom 1991). The redox input (pe) for geochemical calculation was based on the ORP measurement on the leachate and the assumption that sulfur from ICP-SFMS represents sulfate sulfur. The calculated ionic imbalance of the whole dataset is 5.84(± 15.36).

## Results and discussion

### Waste rock characteristics

The waste rock used in this study is andesite. A quantitative X-ray powder diffraction (XRPD) analysis shows that the main minerals in the waste rock are albite (7.0%), biotite (0.48%), microcline (6.7%), muscovite (12.3%), pyrite (0.61%), and quartz (36.3%). Clays, which are common weathering products of rock-forming minerals, i.e., chlorite (10.9%) and smectite (25.7%), are also present (Table 3 in the Appendix). In general, the rocks are devoid of readily soluble minerals capable of neutralizing pH, e.g., calcite (CaCO_3_) or dolomite (CaMg(CO_3_)_2_). However, gangue silicate minerals (e.g., chlorite) and the dissolution of this reactive silicate may provide notable amounts of alkalinity and should be accounted for (Miller et al. 2010).

Analysis by ICP-SFMS and ICP-AES (ALS Scandinavia, Luleå) reveals the rock samples’ chemical composition (Table 2), which consists of 11% S, 9% Al, 13% Fe, and 60% Si, among other elements. Trace elements detected are 19 ppm Co, 38 ppm Cu, 925 ppm Mn, and 8.7 ppm Ni, among others.

**Table 2:** Waste rock chemistry based on ICP-SFMS

Major elements		Trace elements	
Element	wt%	Element	ppm
Al	8.81	As	1.82
Ca	0.43	Co	19.02
Fe	13.20	Cu	37.62
K	3.81	Mn	924.47
Mg	2.08	Ni	8.71
Na	0.25	Pb	4.02
S	10.82	Te	4.39
Si	59.68	Zn	178.68

**Fig. 2:**
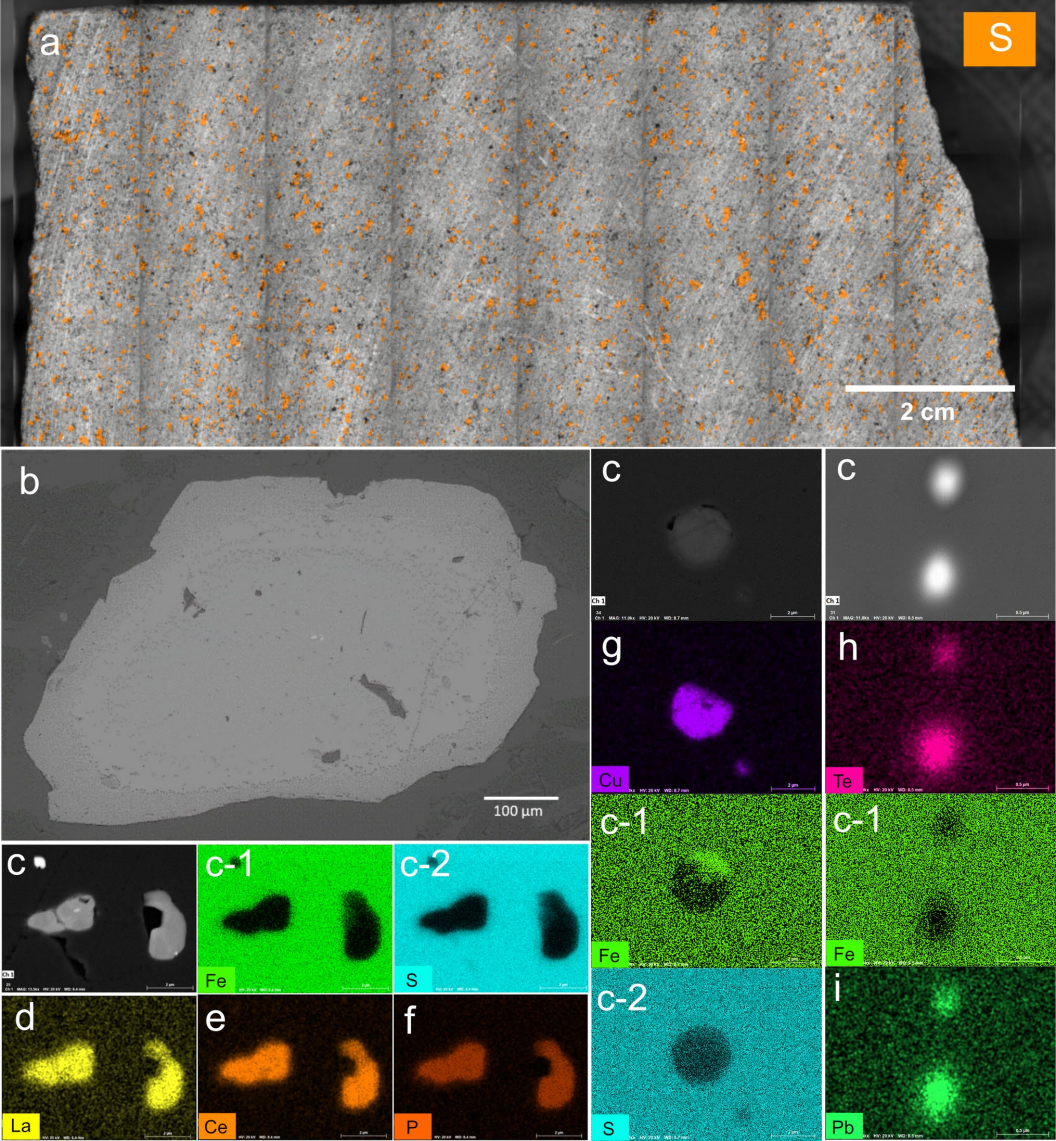
Sulfur distribution in the waste rock by µ-XRF (**a**), backscattered electron (BSE) photomicrograph of pyrite (**b**), trace elements in pyrite (**c**, c-1, c-2), and its corresponding maps of La (**d**), Ce (**e**), P (**f**), Cu (**g**), Te (**h**), and Pb (**i**)

Micro-XRF analysis indicated that the sulfur in the waste rock is disseminated, i.e., dispersed within the rock matrix (Fig. 2a), with size ranging from 0.67 to 1.3 mm and locally forms small patches. Based on the SEM analysis, pyrite is the most abundant sulfide, mainly present as euhedral, cubic crystals (Fig. 2b). The trace elements hosted in the waste rock were examined using various methods including µ-XRF, SEM, and LA-ICP-MS. Based on µ-XRF analysis, it was observed that all Co is hosted in pyrite; Cu coexists with Zn, whereas Mn coexists with Fe. SEM analysis shows that pyrite, as the most abundant sulfide, hosts trace elements such as light REE (Ce, La), Cu, Pb, and Te (Fig. 2). LA-ICP-MS analysis of the pyrite grains showed that Co content is 134–308 ppm. Pyrite also contains Ni in the range of 20 ppm, 19 ppm Se, and 27–35 ppm Ti. Te and Pb are found together with locally high concentrations of Cu, otherwise not present. This analysis also confirmed that the pyrite can contain a high local concentration of Cu, which can be associated with elevated concentrations of As and Ni and the presence of Zn, Te, and Pb. As is found as single digit ppm level and can be present locally with higher concentration together with Mn and Te. Finally, based on the LA-ICP-MS analysis and µ-XRF, Co is an excellent tracer of pyrite oxidation. The deviation between LA-ICP-MS and ICP-SFMS was due to the whole rock digestion for total chemistry analysis, therefore, diluted. Meanwhile, in LA-ICP-MS, only the concentrations of trace elements in pyrite alone are quantified.

### Silica precipitates

Following the addition of silicate solution in cell D, white precipitates visible to the bare eye formed on top of the waste rock and developed as layers on top of the waste rock with time. No white precipitates visible with bare eyes were observed in other Si-treated cells. As the precipitate formed in cell D, Si from the silicate solution remained in the cell at a more extended time than in other cells. Precipitation of silica on top of the waste rock may require a longer contact time to form a layer that is relatively stable over time.

**Fig. 3:**
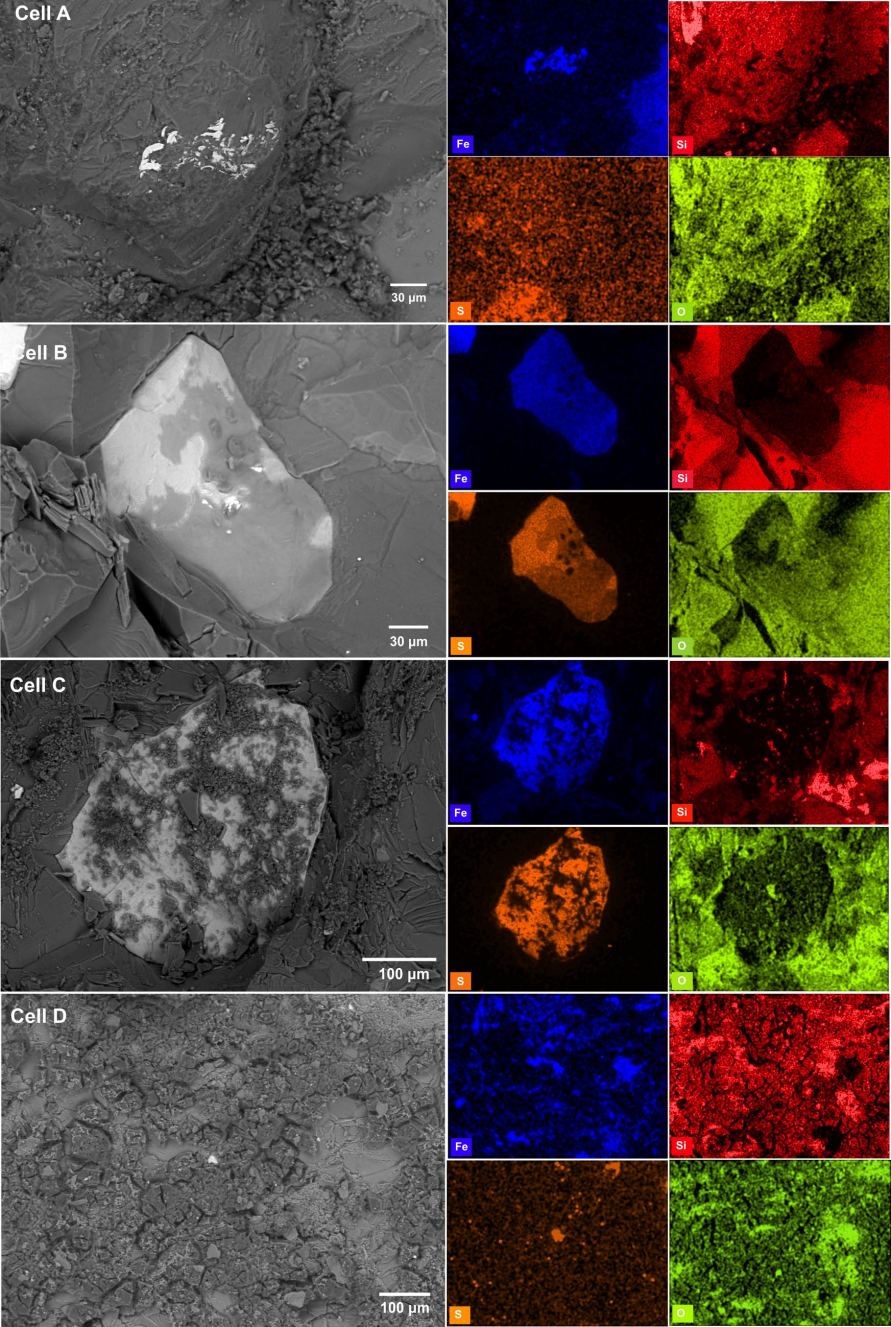
BSE photomicrographs and maps of elemental distribution of the rock sample from all cells at the end of the leaching cycle. Aperture size: 30 µm, accelerating voltage = 20 kV, working distance: 8.5 mm

The chemical composition and morphology of all phases were studied using SEM–EDS on representative rock samples at the end of the leaching period. Additionally, elemental distribution mapping was performed in all identified phases (Fig. 3, right-hand images). In cell A (untreated waste rock), pyrite was intensely oxidized, as shown by the corroded surface and dissolution features (Fig. 3). In all cells with Si-treated rocks, Si was observed to be associated with secondary precipitates, but with differences in the occurrence. In Si-treated waste rock pre-treated with H_2_O_2_ (cell B), pyrite was partly oxidized, as marked by the presence of Fe–O phase (i.e., Fe(oxyhydr)oxide), as opposed to the findings by Kollias et al. (2018), where no iron hydroxide phases were observed as separate precipitates. Silicon was detected within the Fe–O phase by SEM, indicating that Si was absorbed into this phase (Fig. 3). The presence of Si in the amorphous iron (oxyhydr)oxide has been reported to stabilize the passivating layer on pyrite (Fan et al. 2017). Silicate ions form coatings through reaction with the OH groups of ferric hydroxides on the surface of pyrite (Park et al. 2019). It has been understood that silicate species may polymerize at the surface of ferrihydrite (5Fe_2_O_3_·9H_2_O) (Swedlund et al. 2009; Vempati et al. 1990), whereas natural siliceous ferrihydrite may exist in deposits of mine drainage waters (Cismasu et al. 2014). Furthermore, Lee et al. (2011) documented that forming the Fe-silicate complex reduced the activity of oxidants for pyrite, thus reducing pyrite oxidation. In cell C, a precipitate did not form on the rocks despite adding an alkaline silicate solution for three consecutive weeks (Fig. 3).

**Fig. 4:**
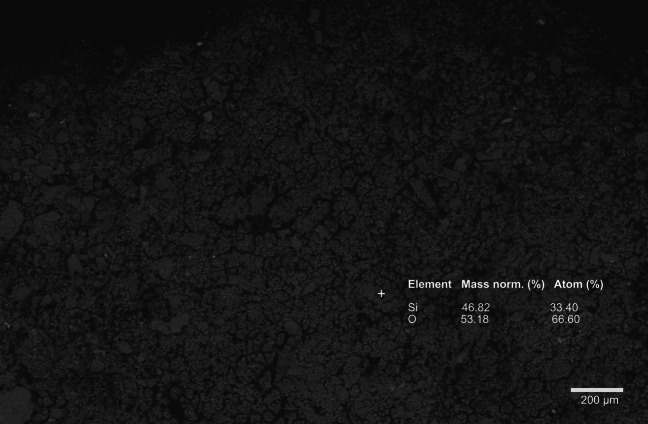
BSE photomicrographs of the formed coating layer in cell D. Magnification: 113 × , aperture size: 30 µm, accelerating voltage = 20 kV, working distance: 8.5 mm. Sample was collected from cycle 21

In cell D, subjected to single Si treatment, a homogeneous layer with a composition corresponding to the stoichiometric ratio Si:O = 1:2 (i.e., silica) precipitated on the surface of waste rock, covering the sulfides and all other phases (Fig. 4). The formed SiO_2_ precipitate by passivating pyrite isolates pyrite surfaces from exposure to oxidants and water (Johnson and Hallberg 2005). This precipitate remained relatively stable following weekly flushing with Milli Q water in each cycle and even after the leaching was terminated 10 weeks after treatment in cell D. The formation of chemically insoluble, inert, and protective surface coating on the pyrite surfaces is desired for AMD control (Fan et al. 2021). However, future studies are required to test the stability of the formed coating under exposure to actual mining environments. Silica precipitate in cell D conforms with the finding by Kollias et al. (2018), whereby pyrite treated with higher Si concentration (i.e., > 0.1 mM Si), Si tends to form stable SiO_2_ precipitates, in comparison to 0.1 mM Si where it seems to favor the formation of Fe oxyhydroxides and adsorbed silicate species (Kollias et al. 2018). The generally approved mechanism for silica precipitation is the polymerization of monosilicic acid (i.e., soluble form of silica, Si(OH)_4_) to form silicate oligomers which often occurs spontaneously at concentrations exceeding 100–200 ppm, followed by subsequent dehydration to form silica (Dyer et al. 2010; Iler 1979).

**Fig. 5:**
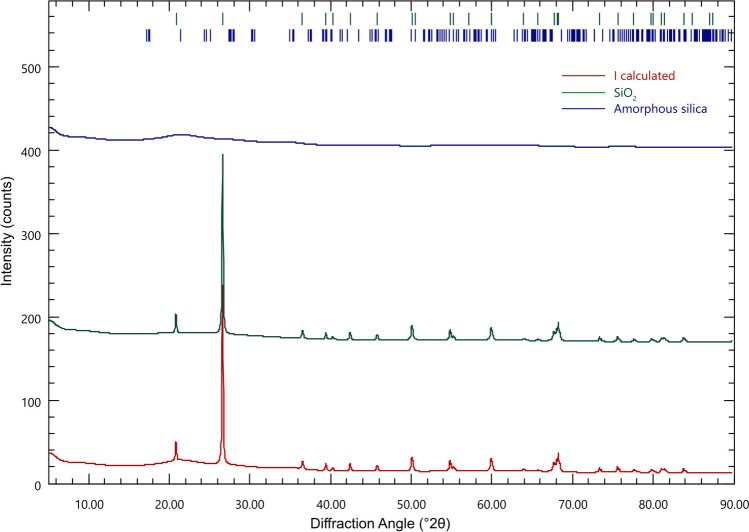
XRD pattern of the formed precipitate in cell D

Based on quantitative XRD analysis (Fig. 5), the formed precipitate comprises 75.40 wt% of amorphous silica and 24.60 wt% of more crystalline silica. The precipitate is composed of 46.74 wt% Si and 53.26 wt% O, which results in the stoichiometric ratio of Si:O = 1:2. It is possible that a longer contact time between the alkaline silicate solution and the waste rock allowed for evaporation and higher concentration of silica, which further led to the polymerization of silica to a more stable, crystalline state as evidenced by the silica layer to remain longer on top of the waste rock longer compared to other treatment. Several months after the leaching was terminated, a watering test was conducted to evaluate the stability of this layer. The hydrated silica layer remained intact based on the SEM analysis, with the EDS showing that the elemental composition of Si:O = 1:2.

### Leaching characteristics

**Fig. 6:**
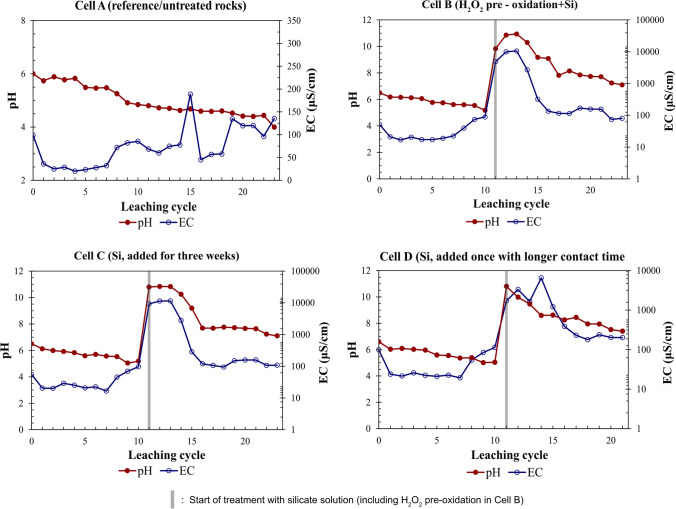
pH and EC profile over weeks in all cells

**Fig. 7:**
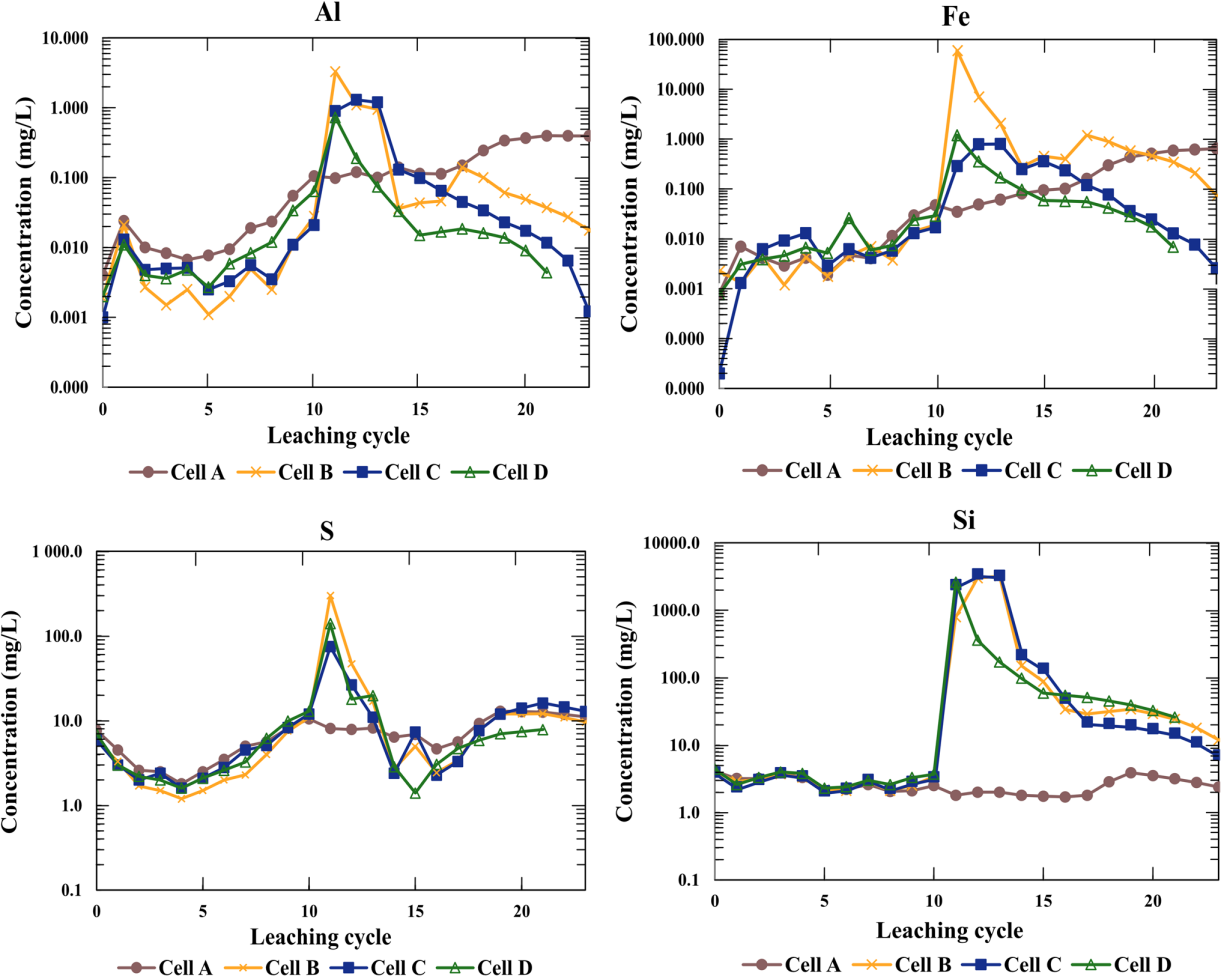
Concentration of major elements in the leachate in the reference cell (untreated waste rock) and Si-treated waste rock

**Fig. 8:**
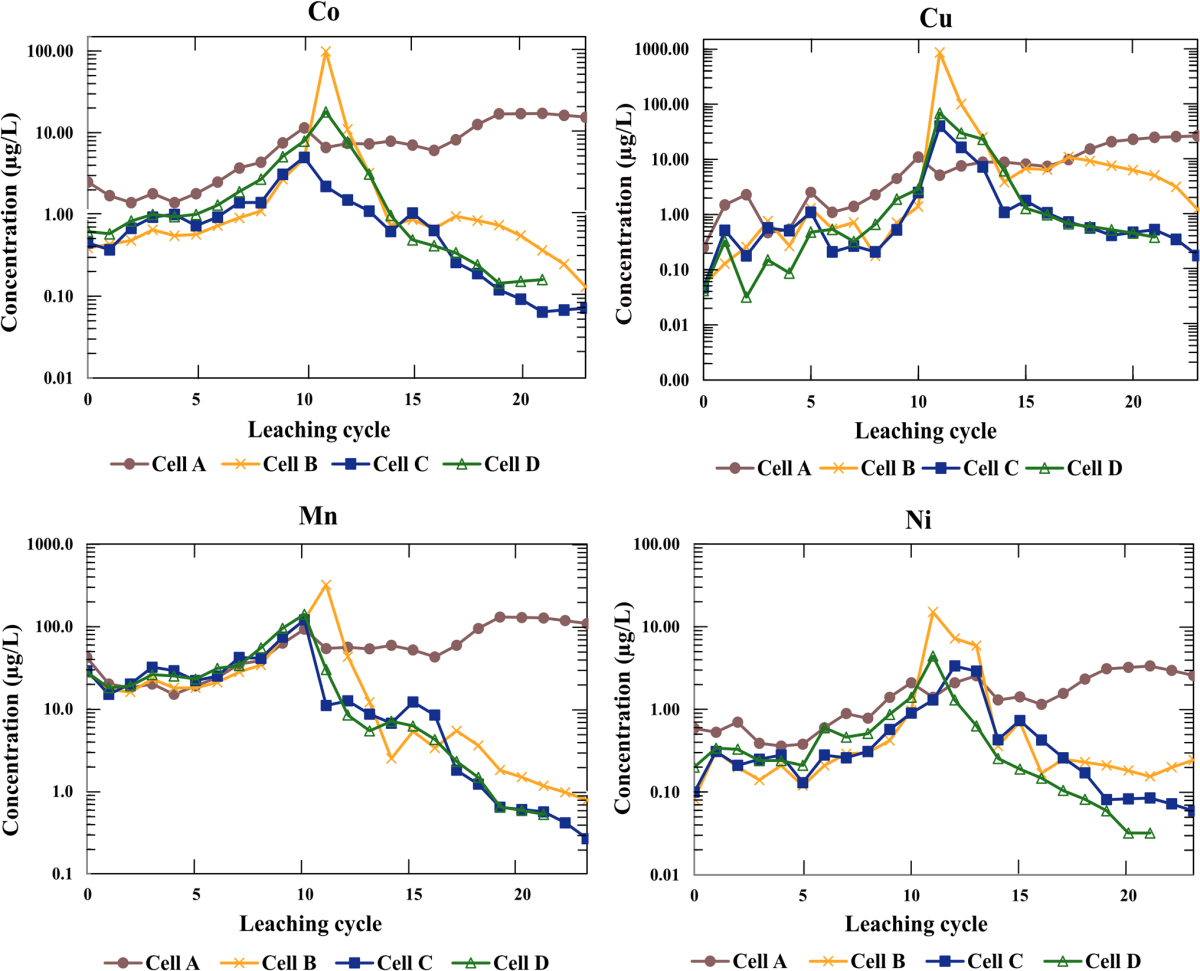
Concentration of trace elements in the leachate in the reference cell (untreated waste rock) and Si-treated waste rock

The following presents and discusses the leaching characteristics of the untreated and Si-treated waste rock with respect to main field parameters (pH, EC, Fig. 6) and selected major and trace elements (Figs. 7 and 8, respectively).

During the first 11 weeks of leaching and before the treatment with silicate solution was initiated, leachate from all cells had a pH, i.e., ≈6. In the cell with untreated waste rock (cell A), pH declined to 4 over time as leaching progressed (Fig. 6), and the concentration of major elements (Al, Fe, S, and Si, Fig. 7) and trace elements (Co, Cu, Mn, and Ni, Fig. 8) in cell A increased progressively. These observations indicated ongoing pyrite oxidation in the untreated waste rock. Cobalt is hosted principally in pyrite and, therefore, a good fingerprint for ongoing pyrite oxidation, confirmed by the elevated release of Co from the leachate in cell A (Fig. 8; Fig. 11 in the Appendix).

The concentrations of major and trace elements in the leachate peaked simultaneously with pH and EC peaks and thereafter decreased over time. In all Si-treated cells, the leachate pH and EC peaked upon adding Si solution, reaching around 10 and 11 mS/cm, respectively, consistent with the alkaline pH and the high dissolved Si and Na concentrations of Si solution. As leaching continued, the pH in all Si-treated cells decreased steadily and stabilized until the end of the leaching period, but both pH and EC remained higher until the end compared to the start of the experiment (Fig. 6). At the end of the leaching period, in all Si-treated cells, the leachate pH stabilized around 7, and the concentrations of major (Al and Fe) and trace elements in pyrite (Co, Cu, Mn, Ni) were lower compared to cell A. The slow pH decrease and lower release of metals in all treated waste rock is attributed to neutralization released upon addition of alkaline silicate solution or surface passivation of pyrite. The pre-oxidized waste rock (cell B) systematically showed the most prominent release of Al, Fe, and S to the leachate than Si-treated cells without H_2_O_2_ pre-oxidation (cell C and cell D) (Fig. 7). No apparent differences between the leaching characteristics from cell C and cell D, i.e., waste rock subjected to multiple and single Si treatment, respectively, were observed. Leaching characteristics of the waste rock treated in different ways in cells B, C, and D showed some differences and similarities. Firstly, peaking, with the highest concentrations of elements, was systematically observed in cell B than in cell C and cell D (Figs. 7 and 8). The concentrations of most major and trace elements showed a decreasing trend weeks after treatment. Cell B showed some exceptions, including S, a somewhat ambiguous trend, and higher variability than those observed in other Si-treated cells (C and D) (Fig. 7; Fig. 8; Fig. 11 in the Appendix). Impurities of the reagents used in the treatments may have played a negligible role in the peaking concentrations (Table 4 in the Appendix); thus, it seems that the pre-treatment with H_2_O_2_ appears not only to result in dissolution of pyrite (Eq. 2) but also to indirect dissolution of other phases, e.g., rock-forming silicates (Eq. 3; Eq. 4), hence releasing several major and trace elements into the leachate. All Si-treated cells generated and maintained a neutral pH in the leachate, with no signs of accelerated sulfide oxidation at the end of the leaching cycle.

The likely explanation for the peaking major and trace cation concentrations upon Si addition is their solubilization due to alkaline pH, and in the case of cell B, the oxidation of pyrite and dissolution of rock-forming minerals upon H_2_O_2_ treatment. Upon addition of H_2_O_2_, dissociation of H_2_O_2_ in water released O_2_ (Eq. 1) and led to elevated concentration of dissolved O_2_ in the solution in cell B. Due to a higher concentration of available oxidants, on-going pyrite oxidation accelerated (Eq. 2), releasing more protons (H^+^) into the solution. Acidity (H^+^) is consumed during the weathering process of rock-forming silicates, for example, albite (NaAlSi_3_O_8_) (Eq. 3) and relatively reactive chlorite (Miller et al. 2010) (Eq. 4), as reflected in the release and peak concentration of Al in the leachate following H_2_O_2_ addition (Fig. 7).

1 $${H}_{2}{O}_{2}\to {H}_{2}O+\frac{1}{2}{O}_{2}$$

2 $$Fe{S}_{2}+\frac{7}{2}{O}_{2}+{H}_{2}O\to {Fe}^{2+} +2{H}^{+}+2{SO}_{4}^{2-}$$

3 $$NaAl{Si}_{3}{O}_{8}+4{H}^{+} +4{H}_{2}O\to {Na}^{+} +{Al}^{3+} +3{H}_{4}Si{O}_{4}$$

4 $$\begin{array}{c}(Mg,Fe{)}_{5}Al({Si}_{3}Al){O}_{10}(OH{)}_{8}+\left(21-x\right){H}^{+}+\left(1.25-0.25x\right){O}_{2}\to \\ x{Mg}^{2+}+\left(5-x\right){Fe}^{3+}+2{Al}^{3+}+3{H}_{4}Si{O}_{4}+(8.5-0.5x){H}_{2}O\end{array}$$

**Fig. 9:**
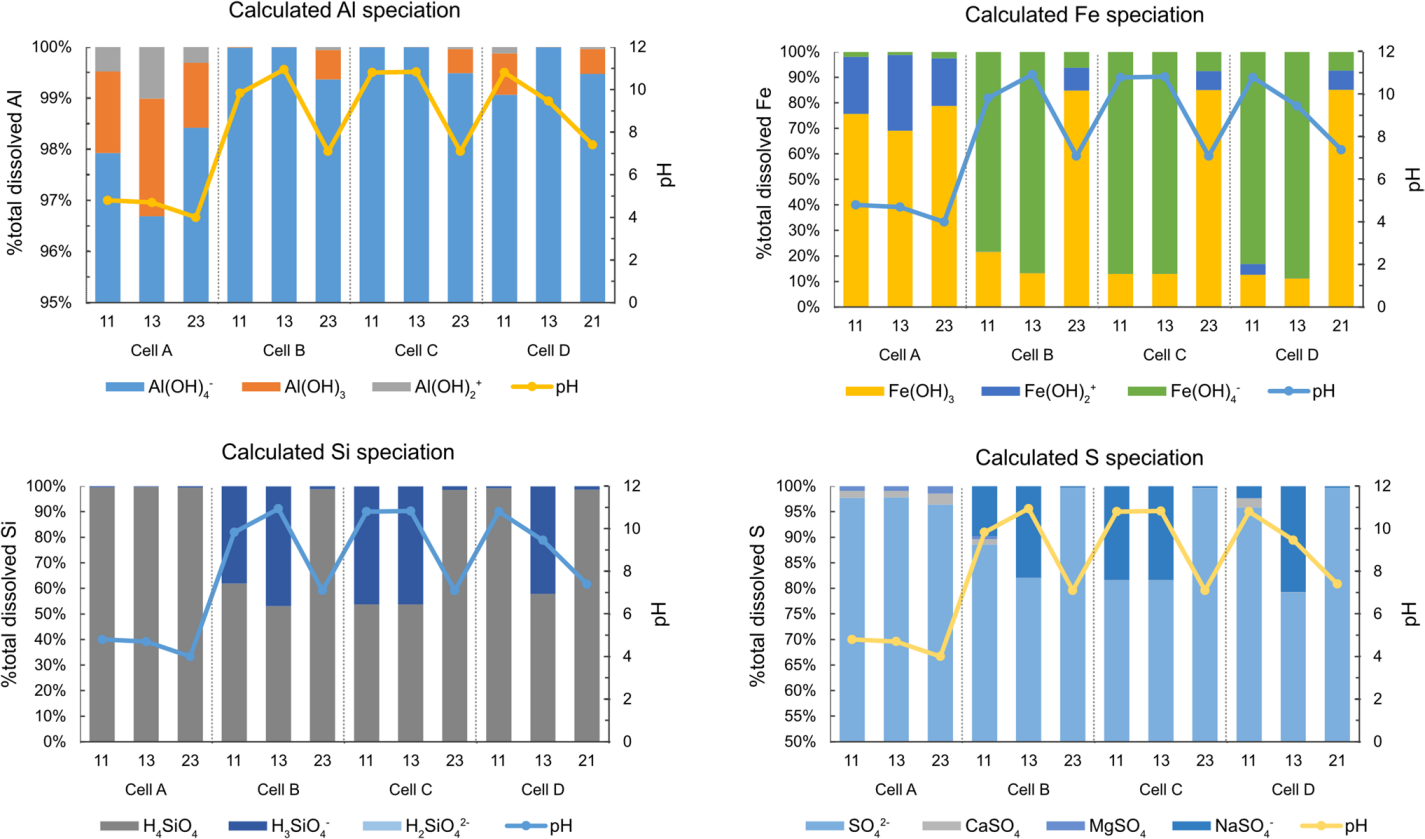
Distribution of aqueous Al, Fe, Si, and S species in the leachates of Si-treated waste rock following the treatment (leaching cycles 11 and 13) and at the end of the leaching cycle, compared to the reference cell

In alkaline pH (≈ 10), Al and Fe may solubilize due to the formation of anionic species Al(OH)_4_^−^ and Fe(OH)_4_^−^ (e.g., Bhattacharya 2013). Geochemical calculations of aqueous speciation in the leachate upon Si treatment confirmed that the Al chemical species in all Si-treated cells (cells B, C, D) are Al(OH)_4_^−^, accounting for nearly 100% of the total dissolved Al. Without any treatment, 97–98% of the total dissolved Al was present as Al(OH)_4_^−^ while the remaining 2% were in the form of Al(OH)_3_ and Al(OH)_2_^+^. In the case of Fe, the main aqueous Fe species in all Si-treated cells was Fe(OH)_4_^−^, whereas Fe(OH)_3_ was the dominant aqueous Fe species in the untreated waste rock (Fig. 9). Hence, it is likely that the solubilization of Al and Fe upon the addition of silicate solution rendered the Al and Fe concentrations higher in the leachate. H_3_SiO_4_^−^ was the main Si species in highly alkaline pH (≈ 10–11), resulting from the dissociation of H_4_SiO_4_. As the pH decreased and stabilized to a circumneutral level, H_4_SiO_4_ became the main Si species (Fig. 9). A similar mechanism is a likely explanation for the peaking concentrations S and other cations, too. The opposing trend of S in all Si-treated cells, compared to the typical decreasing trend as leaching continued after Si treatment, may also arise from the aqueous speciation and enhanced S solubility due to treatment with alkaline Si solution. Based on the aqueous speciation calculations, dissolved sulfur existed as sulfate (SO_4_^2−^) and soluble MgSO_4_ and KSO_4_ species in the leachate from the untreated waste rock. In comparison, SO_4_^2−^ and NaSO_4_ predominated in the leachate following the addition of alkaline silicate solution, whereas nearly all sulfur was present as sulfate in the leachate of all Si-treated waste rock at the end of the leaching cycle.

**Fig. 10:**
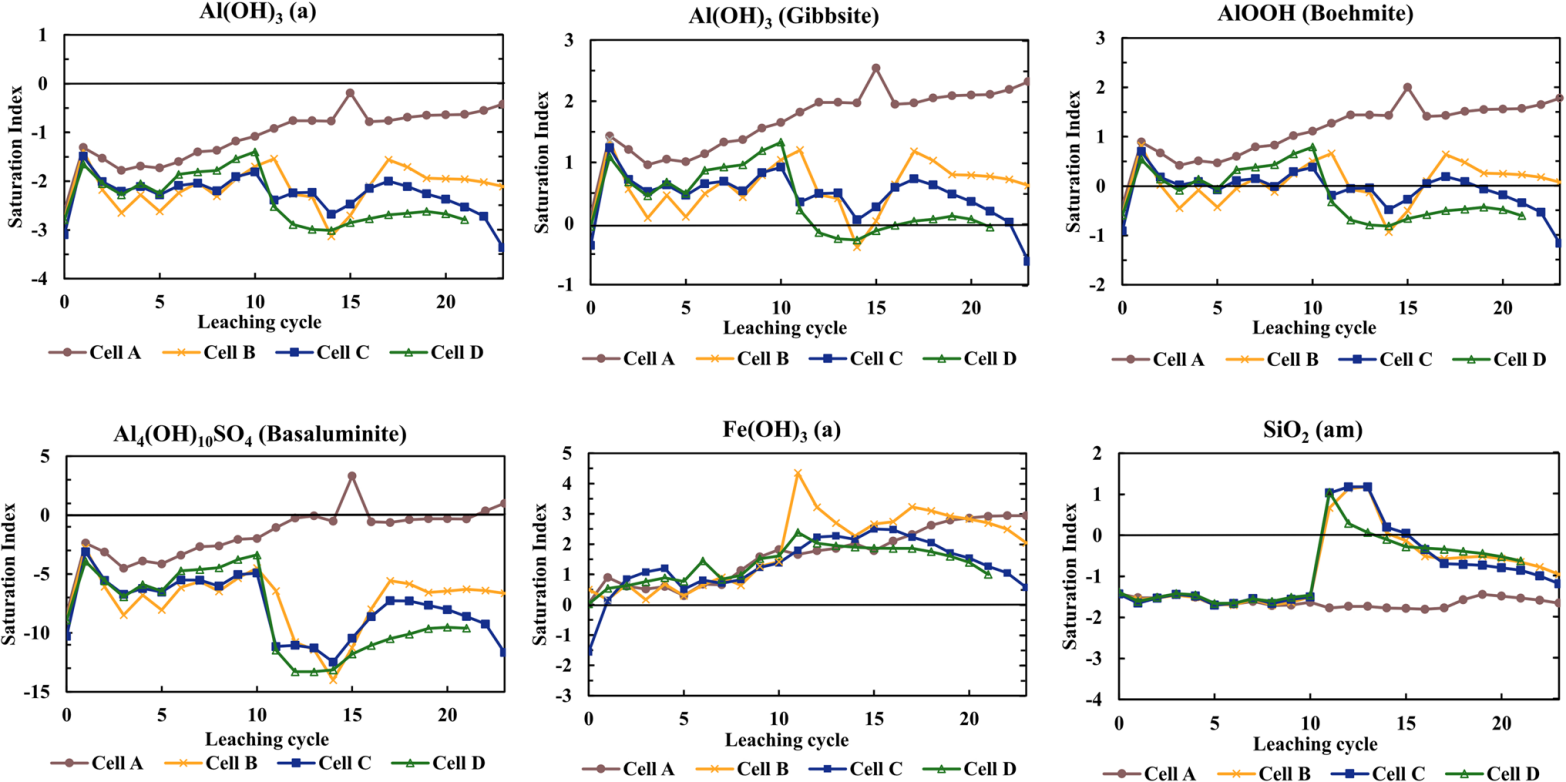
Saturation index (SI) of secondary minerals calculated using PHREEQC using WATEQ4F thermodynamic database

The decreasing trend in the concentrations of major (Al, Fe) and trace elements (Co, Mn, Pb) hosted in pyrite and the neutral leachate pH towards the end of the leaching may be an indication of suppressed pyrite oxidation or due to a circumneutral pH environment causing metals and metalloids to be captured in the secondary phases, or even both. When pH is raised, Fe oxy(hydr)oxides may precipitate, and trace elements (e.g., Cu, Mn, Ni, Zn) may be retained in these phases (Shi et al. 2021). The saturation indices (Fig. 10) showed that leachate samples from the Si-treated cells (cells B, C, D) were supersaturated or close to saturation with respect to Fe(OH)_3_ and Al(OH)_3_ following the treatment of waste rock with silicate solution. These phases may have precipitated in Si-treated waste rock, hence immobilizing Fe and Al.

At the end of the leaching period, the mean percent reduction in dissolved elemental concentrations in the leachate from cells B, C, and D, compared to the reference (cell A) is reported as 94.74% ± 3.54% (Al), 98.89% ± 0.90% (Co), 91.79% ± 10.55% (Cu), and 77.85% ± 32.3% (Fe). In cell D, the reduction in sulfur release in the leachate is 75.2%, compared to 39.3% and 41.6% reduction in sulfur release from cell B and cell C, respectively. This result is in good agreement with Lee et al. (2011) that documented the lower sulfate concentration in the solution when pyritic rock samples were treated with H_2_O_2_ and sodium silicate (Lee et al. 2011), 72% reduction in sulfate release in pyritic tailings treated with Na_2_SiO_3_/H_2_O_2_/CH_3_COONa (Kollias et al. 2022), ≈12% (± 22%) to ≈49% (± 24%) reduction in sulfate on a field-scale treatment of silicate solution on a pyritic coal spoil (Vandiviere and Evangelou 1998), 40–45% reduction in sulfidic slates (Van den Eynde et al. 2009), and ≈35% reduction in sulfur release from the nickel-bearing waste rock treated with Na_2_SiO_3_/H_2_O_2_/NaHCO_3_ buffer solution (Roy et al. 2020).

Leachate quality profile, coupled with geochemical calculations, was used to describe geochemical reactions occurring in untreated and silicate-treated waste rock. Saturation indices (SI) computed with *WATEQ4F* for chemical analysis from the leachates are analyzed whether they are at, below, or above saturation with respect to possible secondary minerals.

Figure 10 shows the range of SI values for amorphous SiO_2_, amorphous Fe(OH)_3_ (ferrihydrite), and selected Al-phases, including amorphous and crystalline Al(OH)_3_ (gibbsite), AlOOH (boehmite), and Al_2_Si_2_O_5_(OH)_4_ (basaluminite) over time. The SI confirms supersaturation of amorphous SiO_2_ in all Si-treated cells upon addition of silicate solution, followed by decreasing SI close to saturation, suggesting that it likely precipitated and then controlled Si solubility. In cells B and C, the silica dissolved over time, whereas in cell D, silica remained in the cell longer than in other Si-treated waste rock. Therefore, weeks following treatment, the solution was undersaturated with respect to amorphous silica. Ferrihydrite and gibbsite may have precipitated in all cells, and the former is a controlling phase in cell D. Amorphous Al(OH)_3_ approached saturation and may have controlled the solubility of A in cell A, as opposed to the Si-treated waste rock, whereas boehmite (AlOOH) approached saturation in cell A and cell D, before and after Si treatment for the latter. Basaluminite (Al_4_(OH)_10_SO_4_), a nanocrystalline aluminumoxyhydrosulfate commonly present in areas affected by ARD (Carrero et al. 2017), may have precipitated in cell A. As the pH became more acidic in cell A at the end of the leaching period, precipitation of basaluminite indicated an ongoing pyrite oxidation.

### Prevention of ARD formation by silica treatment in waste rock

Single silicate treatment of waste rock (Cell D) with a longer contact time showed the most promising results in preventing ARD from sulfidic, acid-producing waste, as evidenced by the homogenous SiO_2_ coating observed on pyrite surfaces and the decreasing trends in the release of metals. This coating layer on pyrite likely acted as a protective barrier against further oxidation and could explain the nearly constant pH at the end of the leaching cycle. The formed precipitate in cell D as layers on top of the waste rock might be important in protecting the pyrite from oxidation, as this layer was not leached at the same rate as in other cells. Furthermore, as the precipitate formed, Si from the silicate solution remained in cell D longer than other cells. This study suggested that silica precipitation may require prolonged time to develop on the surface and finally form a stable layer over time. In addition, adding silicate solution at a slower flow rate might also be required to promote a progressive supersaturation buildup without achieving a highly alkaline pH level, since H_4_SiO_4_ remains undissociated at pH values below 9 and it is a precursor to solid, amorphous silica (SiO_2_) (Iler 1979).

It is also worth noting that this study showed that the effect of contact time of solution is an essential factor in the development of silica layer. The contact time of the silicate solution in cell D was more prolonged than in cell B and cell C to provide sufficient time for the buildup of the SiO_2_ coating which may grow thicker and denser over time. Due to a longer contact time between the alkaline silicate solution and the waste rock in cell D, evaporation may have also resulted in higher silica concentration. Once the concentration exceeded 100 ppm, the monosilicic acid either precipitated or polymerized to form silica layer on top of the waste rock (Iler 1979). This study revealed that even in alkaline solution silica precipitation is not immediately complete but requires several days to attain a steady state.

Nonetheless, despite the SiO_2_ formed, it remains uncertain how long the effect of Si treatment may last and whether the formed precipitate is stable under a more acidic environment. The decreasing trend in the release of metals was observed after Si treatment, followed by initially elevated concentrations of some elements. However, the sulfur release did not show a similar trend, which is believed to be partially due to the formation of soluble NaSO_4_^−^ species from the alkaline Si solution. The current study does not unambiguously show whether the decreasing trends in the release of metals are due to the inhibition of the pyrite surface by precipitation of secondary minerals, the maintenance of a circumneutral pH environment created by the Si treatment, or the combination of both. However, pH sustenance to a circumneutral level by silicate solution is also essential because acid pH conditions enhance the mobility of metals, particularly the divalent cations of Cu, Zn, and Mn (e.g., Dold 2017).

**Fig. 11:**
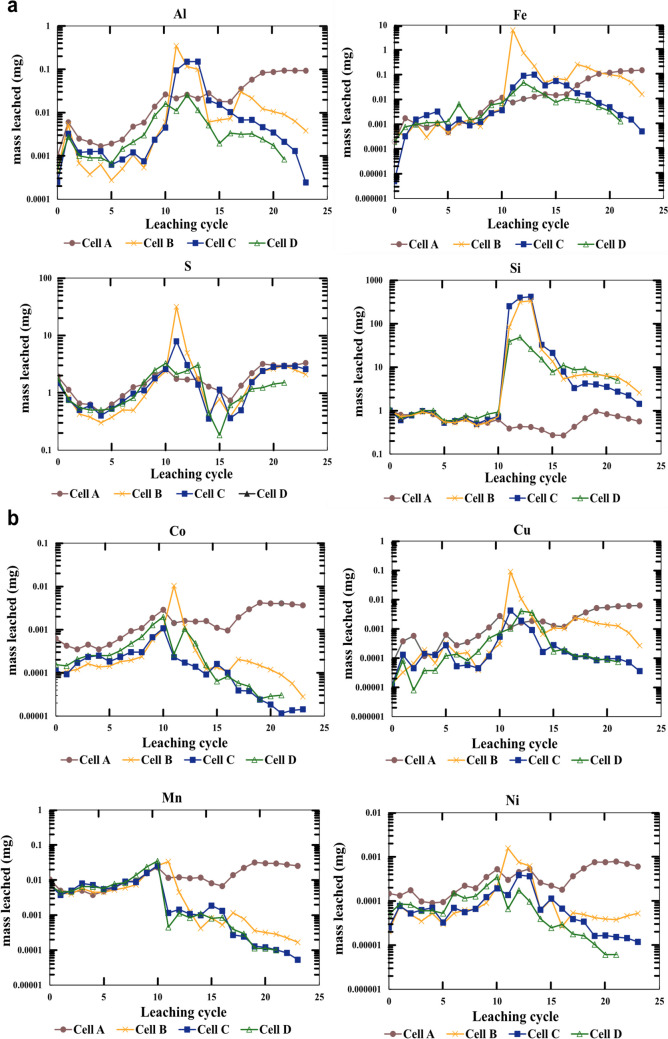
Elemental release of major elements (**a**) and trace elements (**b**) (in mg per leaching cycle) from all cells

Repeatedly adding silicate solution to the pyritic waste rock (cell C) did not improve the overall leachate quality compared to waste rock subjected to single Si treatment (cell D). Furthermore, it did not result in the formation of homogeneous precipitate on the rocks; it only resulted in partial coverage of the pyrite surface. A likely explanation is that the repeated addition of alkaline silicate solution resulted in an alkaline pH > 9 for an extended time, which in turn favors H_4_SiO_4_ dissociation in alkaline solution, giving up H^+^ (Iler 1979). It is understood that the solubility of amorphous silica is little affected by changes of pH in the range 2–9 but increases rapidly as the pH rises above 9 (Iler 1979). The solution pH in cell C was maintained at ~ 10.5 (Fig. 6), where the rate of silica dissolution, which is also catalyzed by hydroxyl ions, becomes significant (Nordström et al. 2011). Repeated addition of silicate solution also resulted in a possible risk of metal mobilization, as shown in elevated concentrations and leached mass of dissolved Al and Fe (Fig. 7; Fig. 11 in the Appendix), persisting over an extended time. Given that repeated treatment did not result in further improvement compared to a single treatment but increased the possible risk for metal leaching due to alkaline pH and additional reagents required, such an approach is not motivated under the circumstances prevailing in this study.

Although the siliceous Fe(oxyhydr)oxide phase is detected in cell B, the pre-oxidation with H_2_O_2_ before Si treatment did not improve the impact of Si treatment compared to treatment with alkaline Si solution only (cells C and D), but rather the opposite. The pre-oxidation step resulted in prominent release of sulfur as well as several major and trace elements (Figs. 7 and 8) upon treatment, as well as showed an ambiguous trend, rather than decreasing trend, in their release to the leachate upon continued leaching. Previous Si inhibition studies involve H_2_O_2_ pre-oxidation prior to the addition of silica solution to form a coating layer on pure pyrite samples (Kollias et al. 2018, 2022; Vandiviere and Evangelou 1998), while the current experiments on waste rock indicate that the pre-oxidation step may not have a positive effect on the leachate quality when applied on to heterogeneous materials, e.g., pyritic waste rock. This finding agrees with the results by Kang et al. (2024), which shows that Fe-silicate-based treatment using H_2_O_2_ pre-oxidation did not properly inhibit sulfidic rock samples, although it appears effective in treating pulverized monomineralic pyrite samples.

Accordingly, the one-time addition of silica on sulfidic waste rock with a longer contact time (cell D) generated near-neutral-alkaline pH with low metal release. Pre-oxidation of waste rock with H_2_O_2_ prior to Si treatment (cell B) or repeated addition of silicate solution to treat the pyritic waste rock (cell C) did not further improve the overall leachate quality or precipitate a protective layer on the pyrite surface in comparison to single Si treatment of waste rock (cell D). In relevance to industrial application, this study serves as a precursor to further experimental work on promoting the formation of Si coatings but with silica-bearing industrial remnants, e.g., slag, or enhanced in situ dissolution of reactive silicate-bearing minerals, e.g., as a potential source of dissolved Si. In the actual mine environment, micas and chlorite, with silica and silicate being the main components, remain stable under mine conditions and maintain the pH (Zhang et al. 2023). Silica is insoluble in acid (pH > 2). It is, therefore, unaffected by the acidification of the mine environment to form a coating on pyrite, despite requiring confirmation to examine the formation and stability of the silica precipitate under long term neutral pH, which further studies in the future must confirm. Finally, this study’s outcome contributes to understanding the geochemical implication of pyrite inhibition by silica, particularly in freshly dumped pyritic waste rock at a waste heap pile in an operating mine site. To warrant applicability in the mine environment, the amount of material required for achieving inhibition of pyrite oxidation prior to field applications must be evaluated in further studies.

## Conclusions

In a small-scale column leaching study, treating sulfidic waste rock with an alkaline silicate solution generated leachate with reduced metal concentrations and maintained a circumneutral pH, as opposed to untreated rock. Sulfide oxidation accelerated in the untreated cell, resulting in low pH and elevated concentration of metals. A single addition of alkaline silicate solution with a longer contact time resulted in homogenous precipitation of silica (SiO_2_) layer on top of the waste rock. The formed silica precipitate remained stable upon weekly dry–wet cycle until the end of the leaching period. In contrast, repeated addition of silicate solution resulted in ongoing dissolution of SiO_2_ at a pH above 10 and led to possible risk of metal mobilization in the solution. Pre-oxidation prior to silicate addition resulted in the formation of a Si-adsorbed Fe(oxyhydr)oxide phase but also rendered the concentrations of metals high in the leachate, presumably indicating the indirect oxidation of pyrite and weathering of rock-forming silicate minerals through acid-consuming reaction. Although previous research suggested the pre-oxidation with H_2_O_2_ prior to silicate treatment on monomineralic pyrite samples to release ferric (Fe^3+^) ions, this does not warrant suitability when applied to complex and heterogeneous samples, such as pyritic waste rock, as it indirectly dissolves partly not only pyrite but also other phases. This study suggested the role of silica in preventing ARD formation, by maintaining a circumneutral pH environment over an extended time and inhibiting the surface of pyrite by precipitation of a secondary mineral in the form of SiO_2_, or a combination of both. Finally, this study serves as a precursor to future experiments to inhibit pyrite surfaces using siliceous industrial by-products or enhanced in situ dissolution of reactive silicate minerals.

## Electronic supplementary material

Below is the link to the electronic supplementary material.

Supplementary file1 (XLSX 281 KB)

Supplementary file2 (DOCX 10892 KB)

## Figures and Tables

**Table 4:**
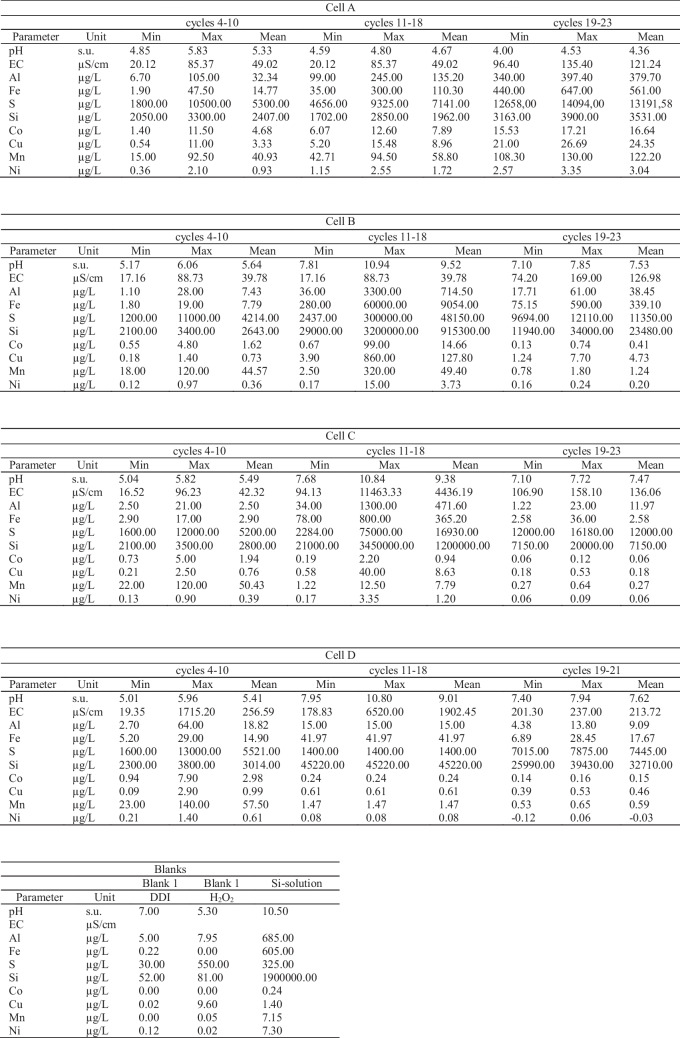
Maximum, minimum, and average values of the measured parameters of leachates from all cells and blank reagents

## Data Availability

The data that support the findings of this study are available within the paper. Should any raw data files be needed in another format, they are available upon request to the corresponding author.

## Appendix

**Table 3:** Mineralogical characterization of the rock samples using XRD

Mineral	Chemical formula	Phase quantity (wt%)	Estimated standard deviation (wt%)
Albite	NaAlSi_3_O_8_	7.00	1.10
Biotite	K(Mg,Fe)_3_AlSi_3_O_10_(F,OH)_2_	0.48	0.38
Chlorite	(Mg,Fe)_3_(Si,Al)_4_O_10_(OH)_2_·(Mg,Fe)_3_(OH)_6_	10.90	1.20
Microcline	KAlSi_3_O_8_	6.70	1.50
Muscovite	KAl_2_(Si_3_Al)O_10_(OH)_2_	12.30	2.30
Pyrite	FeS_2_	0.61	0.20
Smectite	(Mg,Fe^2+^,Fe^3+^)_3_(SiAl)_4_O_10_(OH)_24_H_2_O	25.70	1.70
Quartz	SiO_2_	36.30	1.30
