# Supplementary material for: Prevention of acid rock drainage formation through pyrite inhibition by silica coating
Source: Environ Sci Pollut Res Int. 2025 Feb 27;32(11):6711–31. doi: 10.1007/s11356-025-36131-x (PMC11928364; doi:10.1007/s11356-025-36131-x)
Supplement: Supplementary file 2 — Supplementary file2 (DOCX 10892 KB) [file 11356_2025_36131_MOESM2_ESM.docx]

**Prevention of Acid Rock Drainage formation through pyrite inhibition by silica coating**

Dantie Claudia Butar Butar^1^*, Lena Alakangas^1^, Hanna Kaasalainen^1.2^, Erik Ronne^3^

^1^Applied Geochemistry, Swedish School of Mines, Department of Civil, Environmental and Natural Resources Engineering, Luleå University of Technology, SE-971 87 Luleå, Sweden, [dantie.butar.butar@ltu.se](mailto:dantie.butar.butar@ltu.se)*; [lena.alakangas@ltu.se](mailto:lena.alakangas@ltu.se) ; [hanna.kaasalainen@ltu.se](mailto:hanna.kaasalainen@ltu.se)

^2^Geological Survey of Finland, Vuorimiehentie 5, 02150 Espoo, Finland, [hanna.kaasalainen@gtk.fi](mailto:hanna.kaasalainen@gtk.fi)

^3^Boliden AB, SE-101 20 Stockholm, Sweden, [erik.ronne@boliden.com](mailto:erik.ronne@boliden.com)

**Supplementary Information (SI)**

1. Whole rock samples were subjected to ICP-SFMS for chemical analysis up to 70 elements, normalized to 100%.

| **Major elements** | | **Trace elements** | | | | | |
| --- | --- | --- | --- | --- | --- | --- | --- |
| **Element** | **Wt.%** | **Element** | **ppm** | **Element** | **ppm** | **Element** | **ppm** |
| Al | 8,81 | Ag | 0,15 | Hf | 2,47 | Sb | 0,72 |
| Ca | 0,43 | As | 1,82 | Ho | 0,20 | Sc | 7,71 |
| Fe | 13,20 | Au | 0,04 | Ir | 0,00 | Se | 0,72 |
| K | 3,81 | B | 6,74 | La | 5,97 | Sm | 1,45 |
| Mg | 2,08 | Ba | 572,25 | Li | 8,38 | Sn | 1,36 |
| Na | 0,25 | Be | 0,77 | Lu | 0,08 | Sr | 18,48 |
| S | 10,82 | Bi | 1,10 | Mn | 924,47 | Ta | 0,08 |
| Si | 59,68 | Br | 7,63 | Mo | 1,36 | Tb | 0,17 |
|  |  | Cd | 0,08 | Nb | 1,10 | Te | 4,39 |
|  |  | Co | 19,02 | Ni | 8,71 | Ti | 1340,30 |
|  |  | Cr | 14,06 | Os | 0,00 | Tl | 0,19 |
|  |  | Cs | 0,15 | P | 810,33 | Tm | 0,09 |
|  |  | Cu | 37,62 | Pb | 4,02 | U | 1,28 |
|  |  | Dy | 1,02 | Pd | 0,37 | V | 166,73 |
|  |  | Er | 0,57 | Pr | 1,67 | W | 1,94 |
|  |  | Eu | 0,25 | Pt | 0,00 | Y | 4,96 |
|  |  | Ga | 17,72 | Rb | 8,27 | Yb | 0,55 |
|  |  | Gd | 1,22 | Re | 0,00 | Zn | 178,68 |
|  |  | Ge | 0,71 | Ru | 0,01 | Zr | 94,85 |

**2. Micro XRF**

**a. Major elements**

| 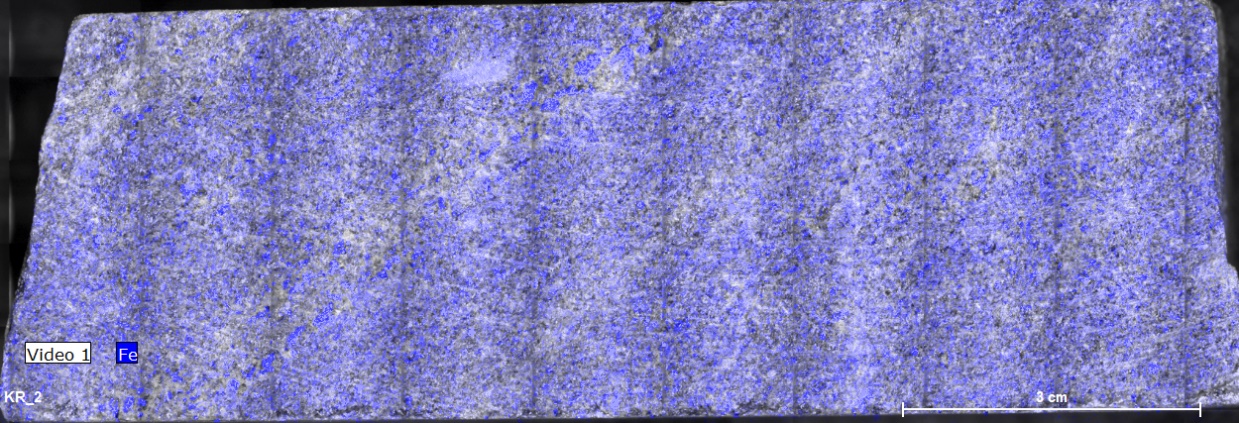 |
| --- |
| 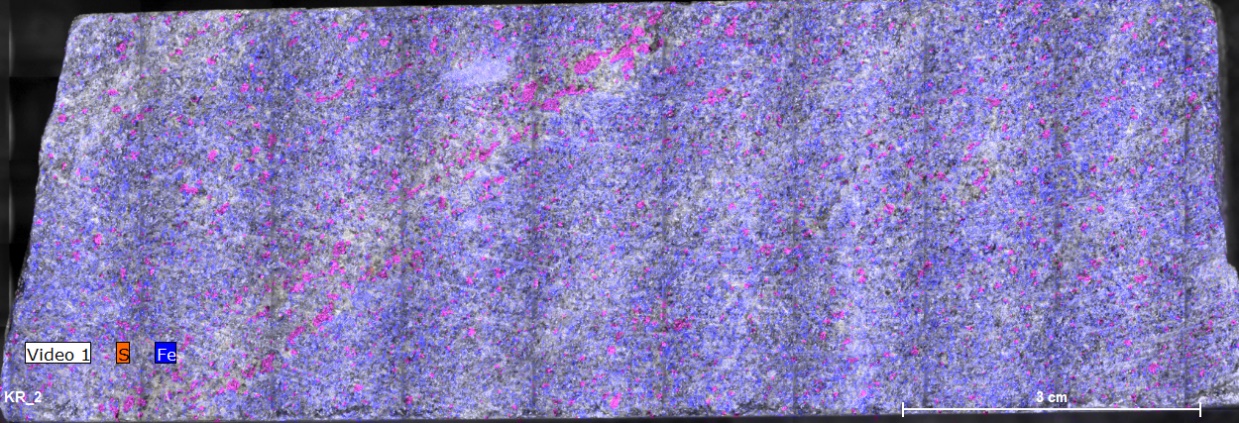 |
| 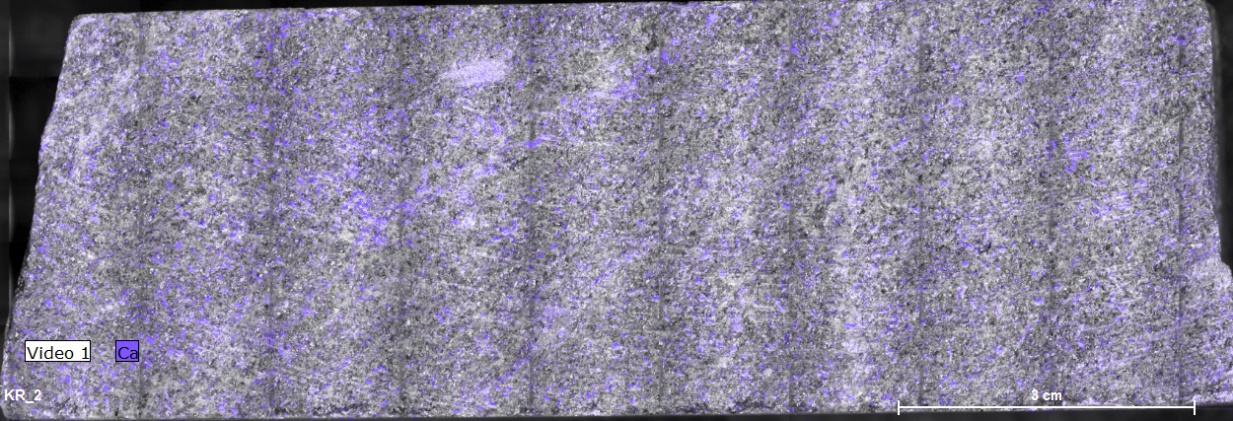 |
| 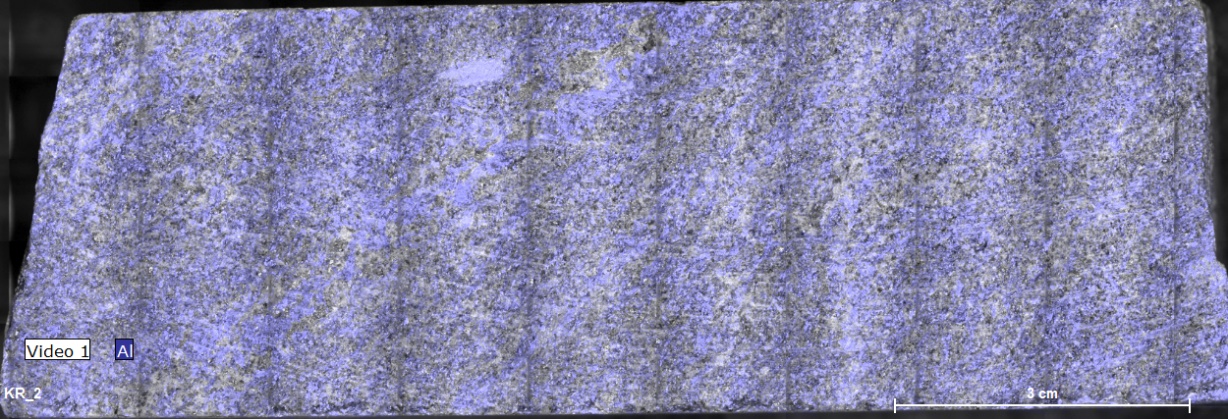 |
| 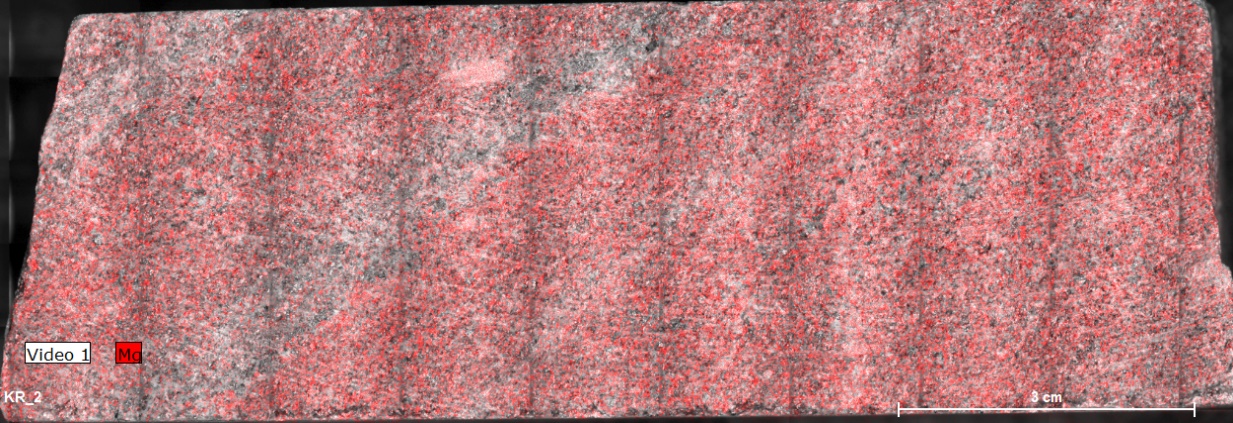 |
| 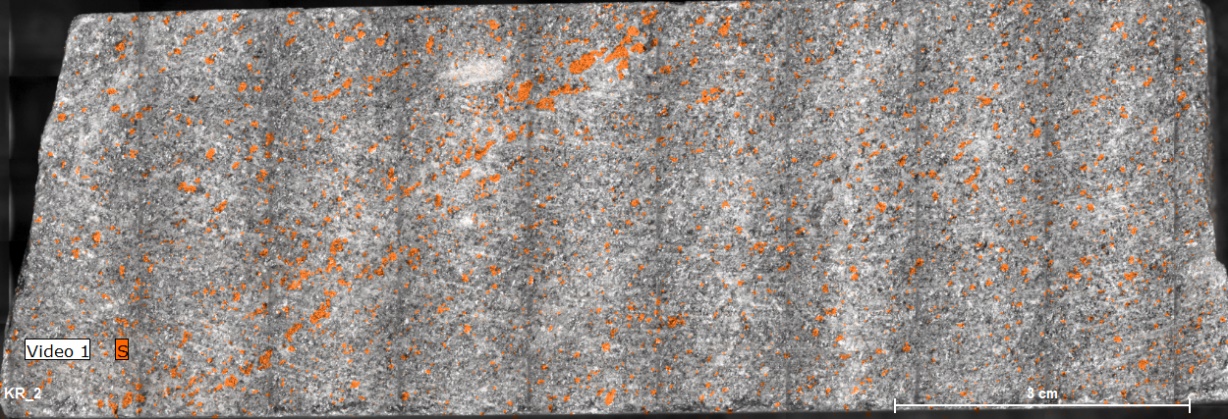 |
| 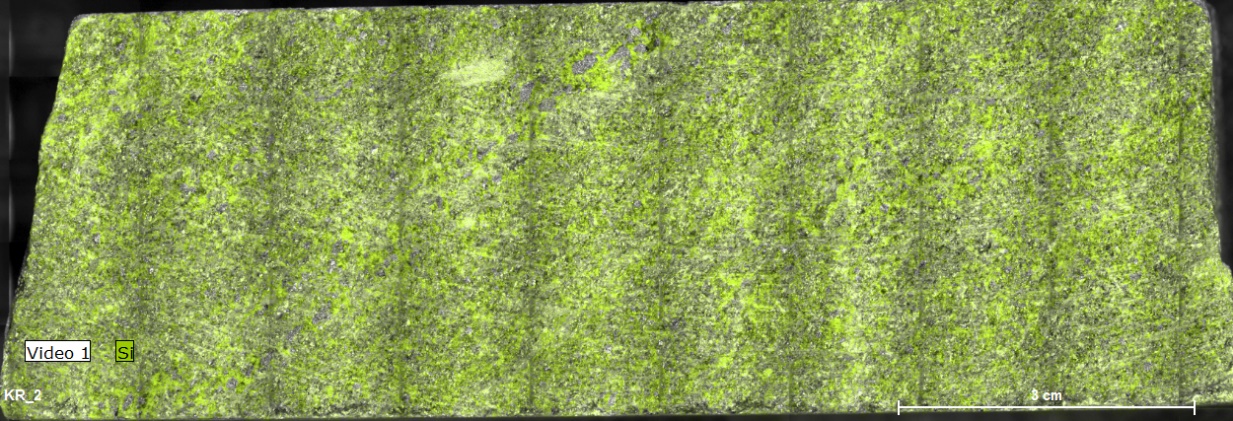 |

**b. Trace elements**

| 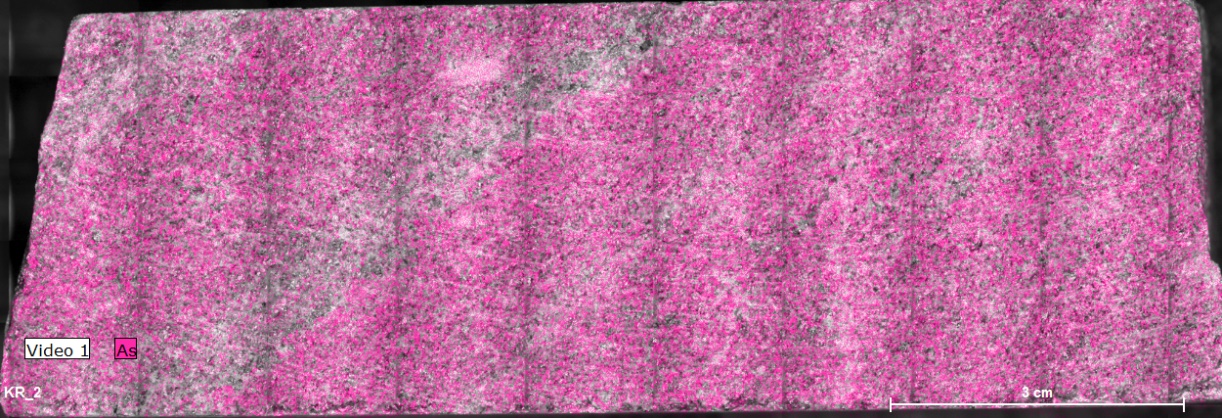 |
| --- |
| 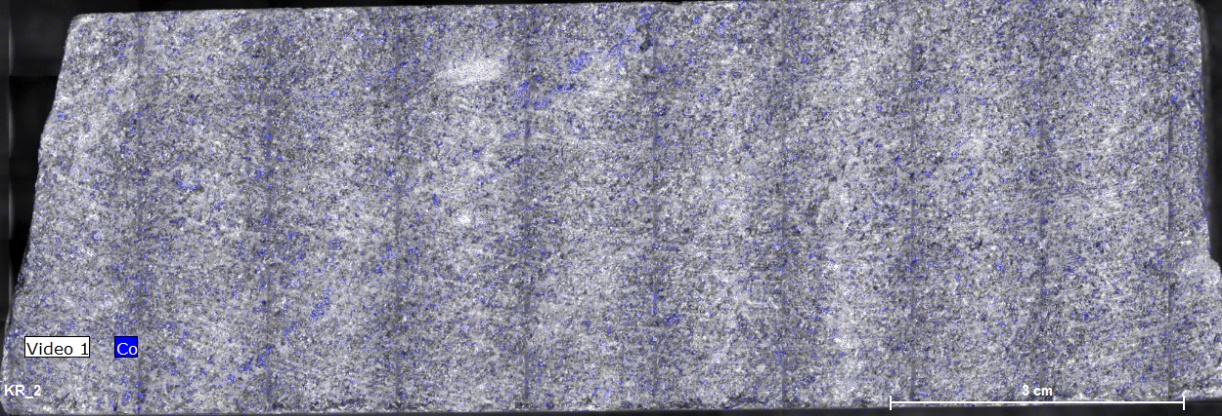 |
| 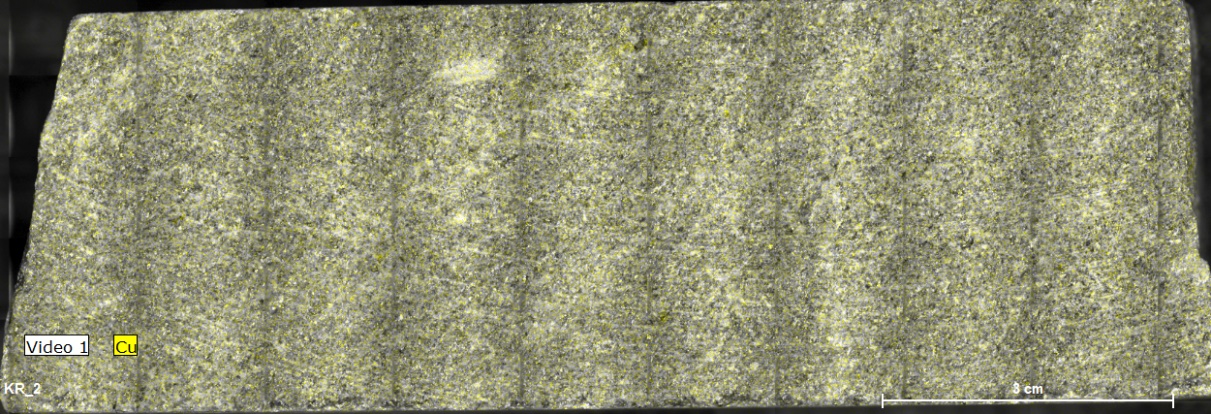 |
| 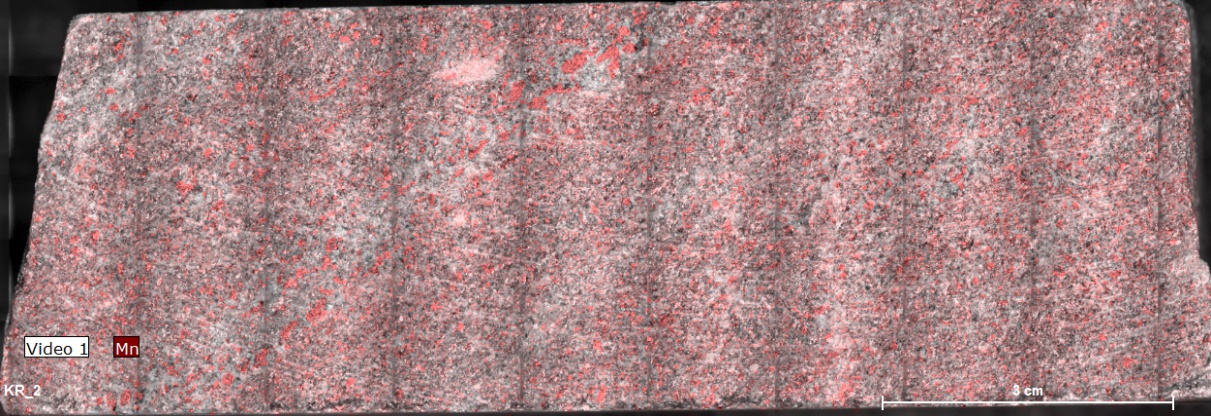 |
| 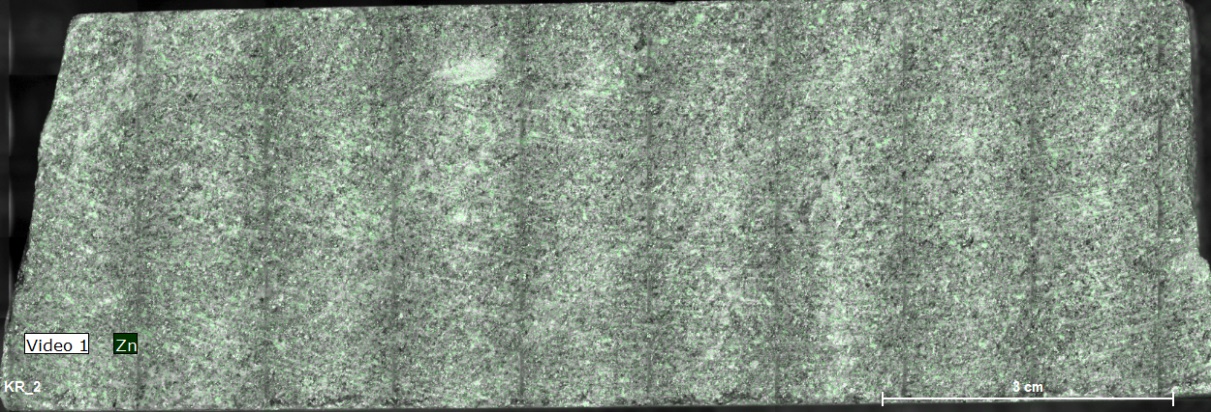 |

3. X-Ray Powder Diffraction (XRPD) of waste rocks


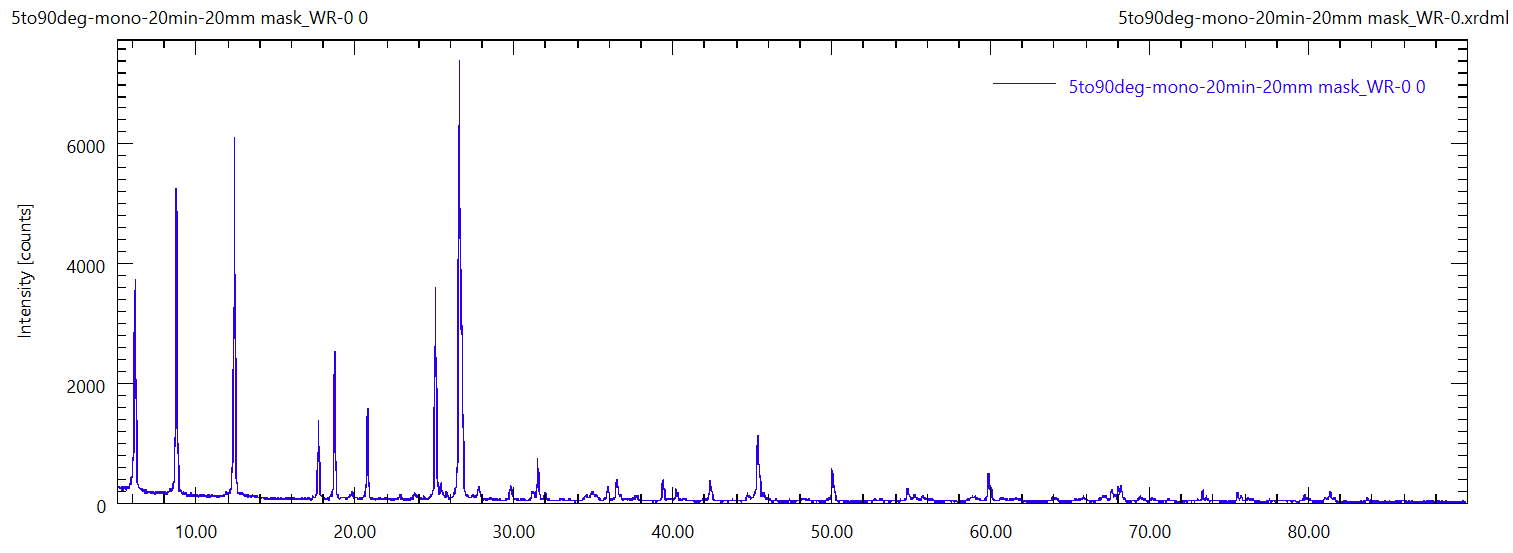


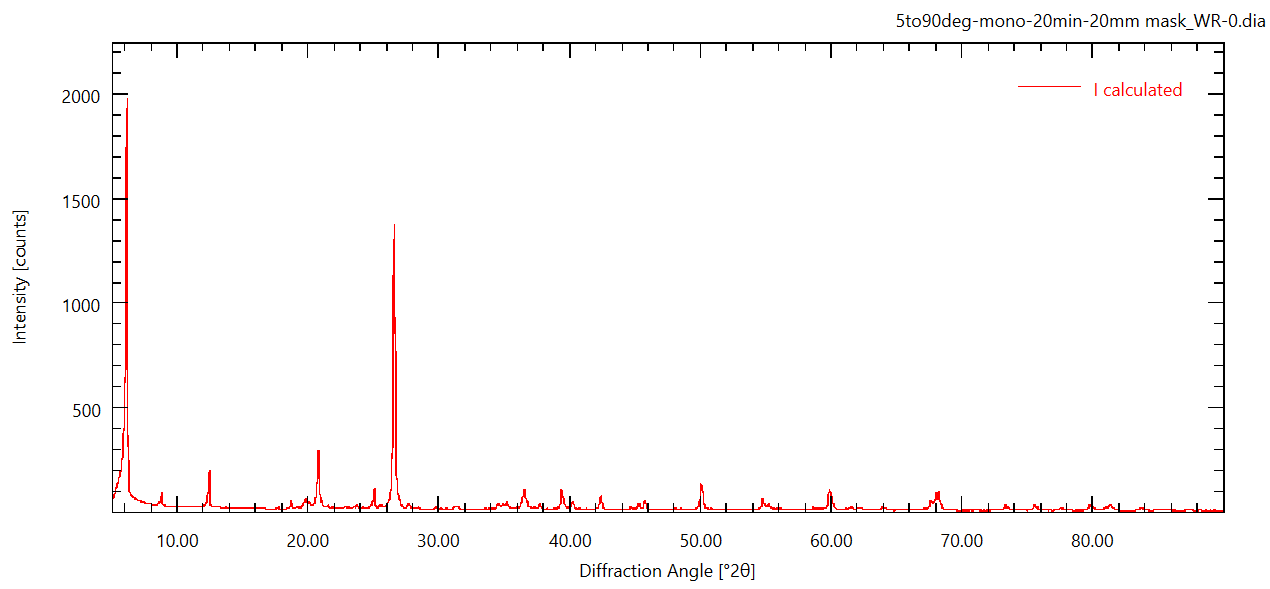


4. Scanning Electron Microscope (SEM)








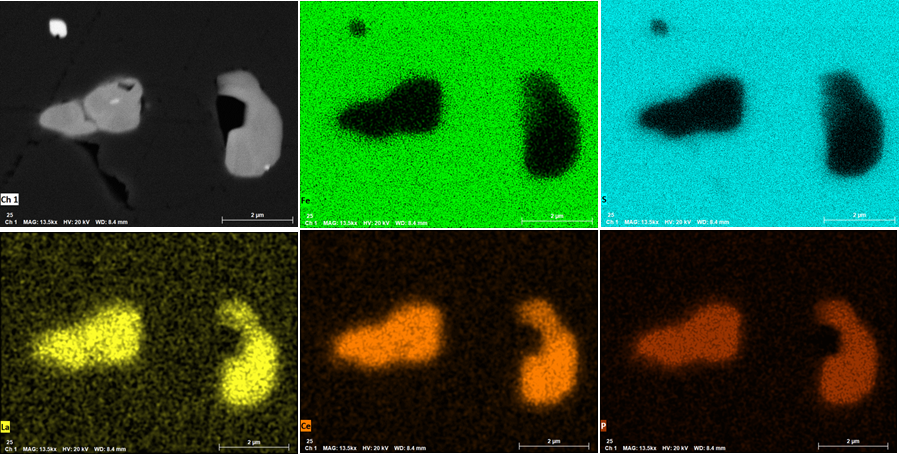




5. Top-view photographs of all cells

a. Leaching cycle 17 (cells A, B, C) and leaching cycle 15 (Cell D)


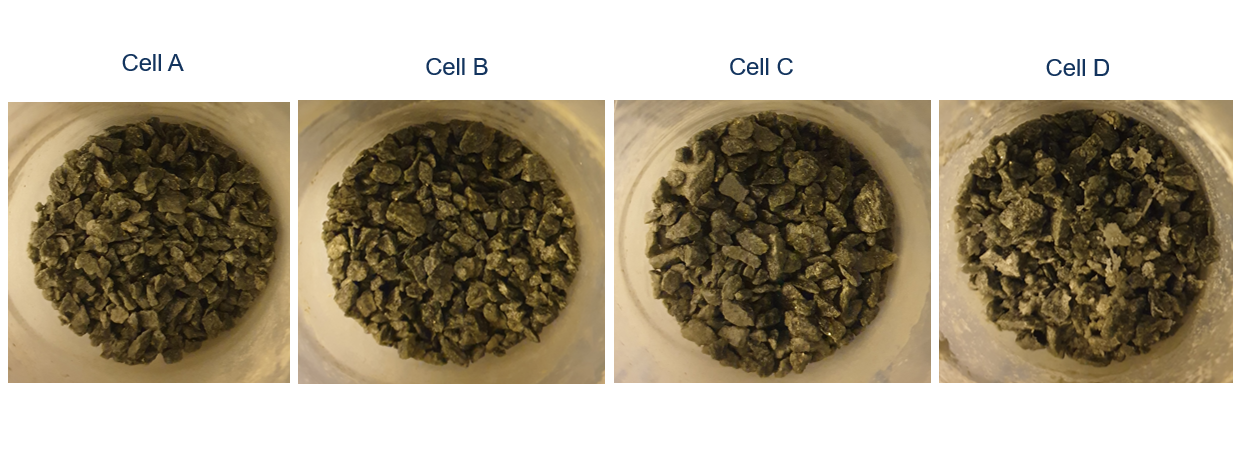


b. Leaching cycle 21 (cells A, B, C) and leaching cycle 19 (Cell D)


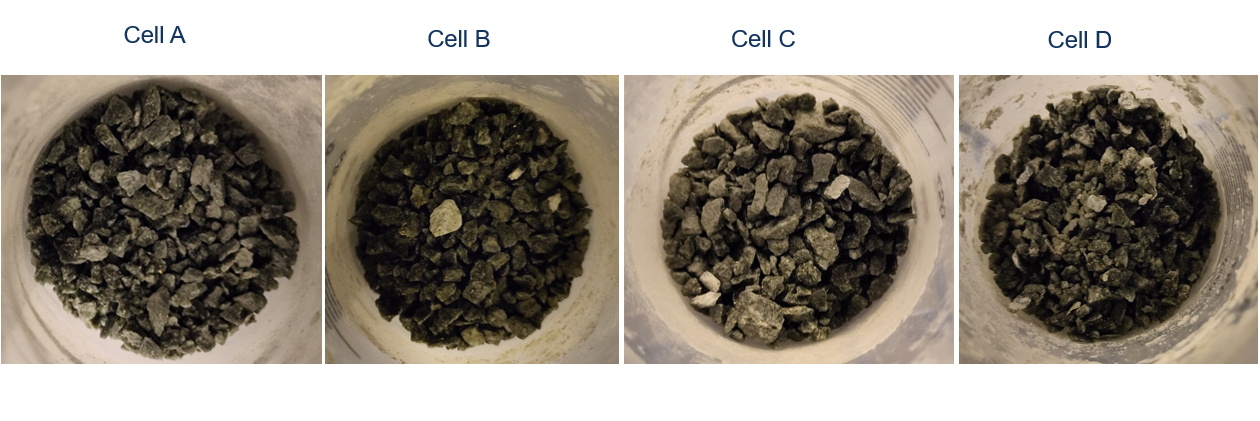


| **Cell A** | | | | | | | | | | | | | | | | | |
| --- | --- | --- | --- | --- | --- | --- | --- | --- | --- | --- | --- | --- | --- | --- | --- | --- | --- |
| **Leaching cycle** | **Major elements** | | | | | | | | **Trace elements** | | | | | | | | |
|  | **Al** | **Ca** | **Fe** | **K** | **Mg** | **Na** | **S** | **Si** | **As** | **Co** | **Cu** | **Mn** | **Ni** | **Pb** | **Te** | **Ti** | **Zn** |
|  | **µg/L** | **µg/L** | **µg/L** | **µg/L** | **µg/L** | **µg/L** | **µg/L** | **µg/L** | **µg/L** | **µg/L** | **µg/L** | **µg/L** | **µg/L** | **µg/L** | **µg/L** | **µg/L** | **µg/L** |
| **Detection limit** | 1.000 | 1.000 | 0.250 | 1.000 | 0.500 | 10.000 | 25.000 | 50.000 | 0.025 | 0.025 | 0.025 | 0.010 | 0.100 | 0.010 | 0.010 | 0.050 | 0.100 |
| **0** | 4.000 | 4600.000 | 0.790 | 3100.000 | 1900.000 | 1000.000 | 7700.000 | 4100.000 | 0.230 | 2.500 | 0.250 | 42.000 | 0.590 | 0.020 | 1.200 | 0.023 | 5.900 |
| **1** | 24.000 | 1800.000 | 7.000 | 1200.000 | 760.000 | 340.000 | 4500.000 | 3200.000 | 0.170 | 1.700 | 1.500 | 20.000 | 0.530 | 0.083 | 1.600 | 0.000 | 5.000 |
| **2** | 10.000 | 1200.000 | 4.200 | 880.000 | 450.000 | 100.000 | 2600.000 | 3200.000 | 0.250 | 1.400 | 2.300 | 18.000 | 0.700 | 0.033 | 2.300 | 0.039 | 5.400 |
| **3** | 8.300 | 1500.000 | 2.900 | 920.000 | 660.000 | 110.000 | 2500.000 | 3900.000 | 0.076 | 1.800 | 0.470 | 20.000 | 0.390 | 0.019 | 2.400 | 0.006 | 3.200 |
| **4** | 6.700 | 1000.000 | 4.200 | 640.000 | 410.000 | 90.000 | 1800.000 | 3300.000 | 0.190 | 1.400 | 0.540 | 15.000 | 0.360 | 0.018 | 2.600 | 0.000 | 3.300 |
| **5** | 7.700 | 1400.000 | 1.900 | 630.000 | 600.000 | 60.000 | 2500.000 | 2100.000 | 0.052 | 1.800 | 2.500 | 19.000 | 0.380 | 0.077 | 1.900 | 0.000 | 4.100 |
| **6** | 9.500 | 1600.000 | 4.700 | 590.000 | 650.000 | 57.000 | 3500.000 | 2200.000 | 0.042 | 2.500 | 1.100 | 24.000 | 0.600 | 0.019 | 2.900 | 0.049 | 5.000 |
| **7** | 19.000 | 2200.000 | 4.100 | 600.000 | 950.000 | 43.000 | 5000.000 | 2600.000 | 0.031 | 3.700 | 1.400 | 35.000 | 0.890 | 0.029 | 4.100 | 0.000 | 10.000 |
| **8** | 23.500 | 2550.000 | 11.500 | 880.000 | 1000.000 | 47.500 | 5650.000 | 2050.000 | 0.090 | 4.350 | 2.300 | 38.500 | 0.781 | 0.017 | 2.750 | 0.000 | 9.000 |
| **9** | 55.000 | 3900.000 | 29.500 | 790.000 | 1600.000 | 40.000 | 8150.000 | 2100.000 | 0.087 | 7.500 | 4.450 | 62.500 | 1.400 | 0.076 | 3.300 | 0.000 | 21.000 |
| **10** | 105.000 | 5700.000 | 47.500 | 725.000 | 2350.000 | 50.500 | 10500.000 | 2500.000 | 0.047 | 11.500 | 11.000 | 92.500 | 2.100 | 0.080 | 3.600 | 0.003 | 45.000 |
| **11** | 99.000 | 3700.000 | 35.000 | 390.000 | 1400.000 | 890.000 | 8100.000 | 1800.000 | 0.090 | 6.600 | 5.200 | 54.000 | 1.400 | 0.110 | 3.800 | 0.140 | 53.000 |
| **12** | 120.000 | 3600.000 | 49.000 | 340.000 | 1500.000 | 58.000 | 7900.000 | 2000.000 | 0.095 | 7.400 | 7.600 | 56.000 | 2.100 | 0.120 | 4.000 | 0.071 | 50.000 |
| **13** | 100.000 | 3450.000 | 61.000 | 285.000 | 1400.000 | 24.500 | 8200.000 | 2000.000 | 0.045 | 7.300 | 8.900 | 53.500 | 2.550 | 0.130 | 5.200 | 0.035 | 25.500 |
| **14** | 140.000 | 3800.000 | 81.000 | 270.000 | 1400.000 | 31.536 | 6400.000 | 1800.000 | 0.083 | 7.900 | 8.900 | 59.000 | 1.300 | 0.100 | 4.000 | 0.029 | 21.000 |
| **15** | 114.687 | 3009.662 | 94.358 | 199.541 | 1182.807 | 83.058 | 6899.030 | 1745.148 | 0.034 | 7.066 | 8.168 | 51.729 | 1.418 | 0.159 | 5.658 | 0.002 | 35.694 |
| **16** | 113.172 | 2940.809 | 101.660 | 195.854 | 1091.793 | 127.256 | 4656.129 | 1701.938 | 0.058 | 6.068 | 7.447 | 42.710 | 1.148 | 0.108 | 3.950 | 0.049 | 18.734 |
| **17** | 150.000 | 3700.000 | 160.000 | 285.000 | 1400.000 | 69.000 | 5650.000 | 1800.000 | 0.100 | 8.200 | 9.950 | 59.000 | 1.550 | 0.080 | 4.000 | 0.047 | 22.000 |
| **18** | 245.000 | 5550.000 | 300.000 | 302.500 | 2100.000 | 89.500 | 9325.000 | 2850.000 | 0.074 | 12.600 | 15.475 | 94.500 | 2.325 | 0.160 | 5.050 | 0.038 | 39.500 |
| **19** | 340.000 | 7400.000 | 440.000 | 320.000 | 2800.000 | 110.000 | 13000.000 | 3900.000 | 0.048 | 17.000 | 21.000 | 130.000 | 3.100 | 0.240 | 6.100 | 0.028 | 57.000 |
| **20** | 368.711 | 7747.671 | 513.653 | 310.234 | 2795.101 | 116.679 | 12829.096 | 3531.373 | 0.041 | 17.104 | 23.051 | 128.360 | 3.223 | 0.318 | 6.685 | 0.048 | 61.702 |
| **21** | 397.421 | 8095.341 | 587.306 | 300.469 | 2790.202 | 123.358 | 12658.192 | 3162.746 | 0.034 | 17.209 | 25.103 | 126.720 | 3.347 | 0.395 | 7.270 | 0.069 | 66.403 |
| **22** | 396.674 | 7210.806 | 617.144 | 285.655 | 2610.314 | 123.816 | 13876.263 | 2779.446 | 0.071 | 16.370 | 25.896 | 117.517 | 2.960 | 0.320 | 5.734 | 0.127 | 58.071 |
| **23** | 395.926 | 6326.272 | 646.982 | 270.841 | 2430.427 | 124.275 | 14094.334 | 2396.145 | 0.108 | 15.531 | 26.689 | 108.315 | 2.574 | 0.245 | 4.198 | 0.185 | 49.738 |

6. Concentrations of selected elements of leachates from all cells and blank reagents (more data on spreadsheet)

| **Cell B** | | | | | | | | | | | | | | | | | |
| --- | --- | --- | --- | --- | --- | --- | --- | --- | --- | --- | --- | --- | --- | --- | --- | --- | --- |
| **Leaching cycle** | **Major elements** | | | | | | | | **Trace elements** | | | | | | | | |
|  | **Al** | **Ca** | **Fe** | **K** | **Mg** | **Na** | **S** | **Si** | **As** | **Co** | **Cu** | **Mn** | **Ni** | **Pb** | **Te** | **Ti** | **Zn** |
|  | **µg/L** | **µg/L** | **µg/L** | **µg/L** | **µg/L** | **µg/L** | **µg/L** | **µg/L** | **µg/L** | **µg/L** | **µg/L** | **µg/L** | **µg/L** | **µg/L** | **µg/L** | **µg/L** | **µg/L** |
| **Detection limit** | 1.000 | 1.000 | 0.250 | 1.000 | 0.500 | 10.000 | 25.000 | 50.000 | 0.025 | 0.025 | 0.025 | 0.010 | 0.100 | 0.010 | 0.010 | 0.050 | 0.100 |
| **0** | 1.900 | 3300.000 | 2.300 | 1800.000 | 1500.000 | 840.000 | 5900.000 | 4000.000 | 0.290 | 0.390 | 0.053 | 27.000 | 0.082 | 0.007 | 0.530 | 0.018 | 1.600 |
| **1** | 21.000 | 1700.000 | 1.300 | 760.000 | 700.000 | 280.000 | 3300.000 | 2900.000 | 0.300 | 0.430 | 0.130 | 17.000 | 0.310 | 0.027 | 0.700 | 0.000 | 0.280 |
| **2** | 2.700 | 1200.000 | 4.400 | 610.000 | 480.000 | 110.000 | 1700.000 | 3100.000 | 0.320 | 0.480 | 0.260 | 16.000 | 0.200 | 0.023 | 1.000 | 0.025 | 2.100 |
| **3** | 1.500 | 1800.000 | 1.200 | 650.000 | 770.000 | 100.000 | 1500.000 | 3700.000 | 0.210 | 0.650 | 0.760 | 23.000 | 0.140 | 0.012 | 1.300 | 0.007 | 1.900 |
| **4** | 2.500 | 1300.000 | 4.300 | 530.000 | 520.000 | 91.000 | 1200.000 | 3300.000 | 0.300 | 0.550 | 0.270 | 18.000 | 0.210 | 0.006 | 1.500 | 0.030 | 1.300 |
| **5** | 1.100 | 1400.000 | 1.800 | 490.000 | 550.000 | 20.000 | 1500.000 | 2200.000 | 0.089 | 0.570 | 1.300 | 18.000 | 0.120 | 0.028 | 1.100 | 0.000 | 6.900 |
| **6** | 2.000 | 1200.000 | 4.500 | 390.000 | 510.000 | 43.000 | 2000.000 | 2100.000 | 0.140 | 0.730 | 0.550 | 21.000 | 0.210 | 0.026 | 1.400 | 0.020 | 1.500 |
| **7** | 4.900 | 1700.000 | 7.100 | 570.000 | 700.000 | 54.000 | 2300.000 | 2900.000 | 0.180 | 0.910 | 0.720 | 28.000 | 0.290 | 0.030 | 1.800 | 0.000 | 11.000 |
| **8** | 2.500 | 2000.000 | 3.800 | 780.000 | 850.000 | 41.000 | 4000.000 | 2100.000 | 0.130 | 1.100 | 0.180 | 34.000 | 0.300 | 0.008 | 1.100 | 0.430 | 1.000 |
| **9** | 11.000 | 4100.000 | 14.000 | 840.000 | 1700.000 | 54.000 | 7500.000 | 2500.000 | 0.150 | 2.700 | 0.710 | 73.000 | 0.420 | 0.063 | 1.600 | 0.000 | 3.800 |
| **10** | 28.000 | 5900.000 | 19.000 | 820.000 | 2600.000 | 84.000 | 11000.000 | 3400.000 | 0.031 | 4.800 | 1.400 | 120.000 | 0.970 | 0.056 | 1.700 | 0.020 | 38.000 |
| **11** | 3300.000 | 45000.000 | 60000.000 | 1200.000 | 11000.000 | 1300000.000 | 300000.000 | 790000.000 | 3.900 | 99.000 | 860.000 | 320.000 | 15.000 | 24.000 | 170.000 | 120.000 | 150.000 |
| **12** | 1100.000 | 11000.000 | 7100.000 | 1800.000 | 2100.000 | 3900000.000 | 47000.000 | 3000000.000 | 12.000 | 11.000 | 100.000 | 43.000 | 7.200 | 3.600 | 90.000 | 140.000 | 51.000 |
| **13** | 950.000 | 5500.000 | 2100.000 | 1700.000 | 550.000 | 3800000.000 | 17000.000 | 3200000.000 | 6.100 | 3.200 | 25.000 | 12.000 | 5.900 | 1.600 | 93.000 | 180.000 | 9.800 |
| **14** | 36.000 | 360.000 | 280.000 | 210.000 | 93.000 | 320000.000 | 2700.000 | 150000.000 | 1.100 | 0.730 | 3.900 | 2.500 | 0.360 | 0.120 | 32.000 | 22.000 | 2.200 |
| **15** | 43.492 | 203.593 | 455.772 | 99.136 | 203.096 | 125011.725 | 4986.579 | 87737.980 | 2.719 | 0.867 | 6.855 | 5.336 | 0.709 | 0.254 | 45.319 | 19.543 | 5.172 |
| **16** | 46.346 | 147.849 | 399.202 | 38.024 | 124.235 | 31343.729 | 2436.764 | 33881.108 | 1.479 | 0.667 | 6.542 | 3.360 | 0.169 | 0.180 | 10.222 | 9.799 | 1.918 |
| **17** | 140.000 | 220.000 | 1200.000 | 79.000 | 120.000 | 28000.000 | 3400.000 | 29000.000 | 1.400 | 0.950 | 11.000 | 5.400 | 0.250 | 0.550 | 5.900 | 19.000 | 1.400 |
| **18** | 100.500 | 225.000 | 895.000 | 64.000 | 106.500 | 30500.000 | 7700.000 | 31500.000 | 1.200 | 0.845 | 9.350 | 3.600 | 0.230 | 0.420 | 4.750 | 13.800 | 2.000 |
| **19** | 61.000 | 230.000 | 590.000 | 49.000 | 93.000 | 33000.000 | 12000.000 | 34000.000 | 1.000 | 0.740 | 7.700 | 1.800 | 0.210 | 0.290 | 3.600 | 8.600 | 2.600 |
| **20** | 49.052 | 177.127 | 469.496 | 40.162 | 71.469 | 28148.854 | 12055.743 | 29120.008 | 0.667 | 0.553 | 6.410 | 1.482 | 0.182 | 0.288 | 3.401 | 6.769 | 2.039 |
| **21** | 37.104 | 124.255 | 348.993 | 31.325 | 49.939 | 23297.707 | 12111.485 | 24240.015 | 0.335 | 0.365 | 5.120 | 1.163 | 0.155 | 0.286 | 3.202 | 4.938 | 1.479 |
| **22** | 27.408 | 90.015 | 212.070 | 32.580 | 32.201 | 19809.846 | 10902.777 | 18090.484 | 0.226 | 0.248 | 3.181 | 0.971 | 0.199 | 0.174 | 2.175 | 2.860 | 1.153 |
| **23** | 17.711 | 55.776 | 75.147 | 33.836 | 14.464 | 16321.985 | 9694.068 | 11940.954 | 0.118 | 0.130 | 1.242 | 0.779 | 0.244 | 0.062 | 1.147 | 0.783 | 0.828 |

| **Cell C** | | | | | | | | | | | | | | | | | |
| --- | --- | --- | --- | --- | --- | --- | --- | --- | --- | --- | --- | --- | --- | --- | --- | --- | --- |
| **Leaching cycle** | **Major elements** | | | | | | | | **Trace elements** | | | | | | | | |
|  | **Al** | **Ca** | **Fe** | **K** | **Mg** | **Na** | **S** | **Si** | **As** | **Co** | **Cu** | **Mn** | **Ni** | **Pb** | **Te** | **Ti** | **Zn** |
|  | **µg/L** | **µg/L** | **µg/L** | **µg/L** | **µg/L** | **µg/L** | **µg/L** | **µg/L** | **µg/L** | **µg/L** | **µg/L** | **µg/L** | **µg/L** | **µg/L** | **µg/L** | **µg/L** | **µg/L** |
| **Detection limit** | 1.000 | 1.000 | 0.250 | 1.000 | 0.500 | 10.000 | 25.000 | 50.000 | 0.025 | 0.025 | 0.025 | 0.010 | 0.100 | 0.010 | 0.010 | 0.050 | 0.100 |
| **0** | 1.000 | 3600.000 | 0.020 | 1900.000 | 1600.000 | 910.000 | 5900.000 | 4000.000 | 0.310 | 0.450 | 0.047 | 29.000 | 0.100 | 0.005 | 0.420 | 0.000 | 1.900 |
| **1** | 13.000 | 1400.000 | 1.300 | 700.000 | 600.000 | 240.000 | 3000.000 | 2400.000 | 0.260 | 0.370 | 0.520 | 15.000 | 0.310 | 0.029 | 0.670 | 0.100 | 0.610 |
| **2** | 4.800 | 1500.000 | 6.200 | 650.000 | 600.000 | 120.000 | 2000.000 | 3100.000 | 0.210 | 0.680 | 0.180 | 20.000 | 0.210 | 0.049 | 1.500 | 0.095 | 3.800 |
| **3** | 5.000 | 2400.000 | 9.300 | 790.000 | 1000.000 | 110.000 | 2400.000 | 3900.000 | 0.140 | 0.930 | 0.570 | 32.000 | 0.250 | 0.041 | 1.700 | 0.000 | 2.300 |
| **4** | 5.100 | 1900.000 | 13.000 | 590.000 | 770.000 | 83.000 | 1600.000 | 3500.000 | 0.320 | 1.000 | 0.510 | 29.000 | 0.280 | 0.038 | 2.200 | 0.000 | 6.700 |
| **5** | 2.500 | 1500.000 | 2.900 | 510.000 | 660.000 | 20.000 | 2100.000 | 2100.000 | 0.053 | 0.730 | 1.100 | 22.000 | 0.130 | 0.048 | 1.400 | 0.000 | 0.960 |
| **6** | 3.300 | 1600.000 | 6.200 | 480.000 | 600.000 | 72.000 | 2800.000 | 2300.000 | 0.110 | 0.930 | 0.210 | 25.000 | 0.280 | 0.018 | 1.900 | 0.110 | 1.500 |
| **7** | 5.600 | 2500.000 | 4.200 | 620.000 | 1000.000 | 58.000 | 4500.000 | 3100.000 | 0.073 | 1.400 | 0.270 | 42.000 | 0.260 | 0.029 | 2.000 | 0.034 | 6.700 |
| **8** | 3.500 | 2400.000 | 5.800 | 820.000 | 990.000 | 53.000 | 5100.000 | 2300.000 | 0.140 | 1.400 | 0.210 | 41.000 | 0.310 | 0.012 | 1.100 | 0.000 | 5.000 |
| **9** | 11.000 | 4000.000 | 13.000 | 880.000 | 1700.000 | 64.000 | 8300.000 | 2900.000 | 0.010 | 3.100 | 0.530 | 74.000 | 0.570 | 0.051 | 1.500 | 0.000 | 8.100 |
| **10** | 21.000 | 6400.000 | 17.000 | 780.000 | 2500.000 | 83.000 | 12000.000 | 3400.000 | 0.025 | 5.000 | 2.500 | 120.000 | 0.900 | 0.050 | 1.500 | 0.000 | 30.000 |
| **11** | 900.000 | 19000.000 | 290.000 | 2000.000 | 550.000 | 4400000.000 | 75000.000 | 2400000.000 | 17.000 | 2.200 | 40.000 | 11.000 | 1.300 | 2.800 | 290.000 | 42.000 | 25.000 |
| **12** | 1300.000 | 15000.000 | 790.000 | 2100.000 | 310.000 | 4950000.000 | 26500.000 | 3450000.000 | 7.500 | 1.500 | 16.500 | 12.500 | 3.350 | 3.100 | 200.000 | 215.000 | 12.000 |
| **13** | 1200.000 | 9400.000 | 800.000 | 1900.000 | 160.000 | 4000000.000 | 11000.000 | 3300000.000 | 5.400 | 1.100 | 7.300 | 8.600 | 2.900 | 3.200 | 180.000 | 220.000 | 12.000 |
| **14** | 130.000 | 1500.000 | 250.000 | 310.000 | 230.000 | 440000.000 | 2400.000 | 220000.000 | 1.800 | 0.620 | 1.100 | 6.700 | 0.430 | 0.490 | 33.000 | 48.000 | 3.300 |
| **15** | 98.268 | 437.124 | 358.742 | 145.322 | 348.169 | 176914.296 | 7332.457 | 137694.413 | 3.815 | 1.039 | 1.791 | 12.078 | 0.734 | 0.605 | 56.188 | 43.686 | 14.802 |
| **16** | 65.140 | 213.814 | 235.213 | 50.215 | 172.165 | 49582.664 | 2283.769 | 49862.770 | 2.161 | 0.640 | 1.076 | 8.405 | 0.427 | 0.283 | 16.032 | 29.392 | 7.554 |
| **17** | 45.000 | 110.000 | 120.000 | 38.000 | 37.000 | 25000.000 | 3300.000 | 22000.000 | 0.930 | 0.260 | 0.730 | 1.800 | 0.260 | 0.210 | 4.000 | 17.000 | 1.700 |
| **18** | 34.000 | 180.000 | 78.000 | 45.500 | 26.500 | 26000.000 | 7650.000 | 21000.000 | 0.680 | 0.190 | 0.575 | 1.220 | 0.171 | 0.165 | 2.950 | 10.450 | 5.500 |
| **19** | 23.000 | 250.000 | 36.000 | 53.000 | 16.000 | 27000.000 | 12000.000 | 20000.000 | 0.430 | 0.120 | 0.420 | 0.640 | 0.081 | 0.120 | 1.900 | 3.900 | 9.300 |
| **20** | 17.380 | 193.137 | 24.430 | 44.568 | 14.176 | 27704.550 | 14090.842 | 17564.699 | 0.382 | 0.092 | 0.476 | 0.602 | 0.083 | 0.095 | 1.729 | 2.404 | 13.694 |
| **21** | 11.760 | 136.275 | 12.860 | 36.136 | 12.352 | 28409.100 | 16181.685 | 15129.398 | 0.334 | 0.064 | 0.532 | 0.564 | 0.085 | 0.071 | 1.557 | 0.907 | 18.089 |
| **22** | 6.491 | 106.180 | 7.719 | 30.072 | 9.845 | 24378.154 | 14537.708 | 11139.487 | 0.250 | 0.068 | 0.356 | 0.415 | 0.072 | 0.042 | 1.320 | 0.576 | 17.046 |
| **23** | 1.223 | 76.085 | 2.577 | 24.008 | 7.338 | 20347.208 | 12893.731 | 7149.576 | 0.165 | 0.071 | 0.180 | 0.266 | 0.059 | 0.013 | 1.082 | 0.244 | 16.003 |

| **Cell D** | | | | | | | | | | | | | | | | | |
| --- | --- | --- | --- | --- | --- | --- | --- | --- | --- | --- | --- | --- | --- | --- | --- | --- | --- |
| **Leaching cycle** | **Major elements** | | | | | | | | **Trace elements** | | | | | | | |  |
|  | **Al** | **Ca** | **Fe** | **K** | **Mg** | **Na** | **S** | **Si** | **As** | **Co** | **Cu** | **Mn** | **Ni** | **Pb** | **Te** | **Ti** | **Zn** |
|  | **µg/L** | **µg/L** | **µg/L** | **µg/L** | **µg/L** | **µg/L** | **µg/L** | **µg/L** | **µg/L** | **µg/L** | **µg/L** | **µg/L** | **µg/L** | **µg/L** | **µg/L** | **µg/L** | **µg/L** |
| **Detection limit** | 1,000 | 1,000 | 0,250 | 1,000 | 0,500 | 10,000 | 25,000 | 50,000 | 0,025 | 0,025 | 0,025 | 0,010 | 0,100 | 0,010 | 0,010 | 0,050 | 0,100 |
| **0** | 2.000 | 3600.000 | 0.770 | 2300.000 | 1600.000 | 990.000 | 7000.000 | 4400.000 | 0.390 | 0.620 | 0.042 | 28.000 | 0.200 | 0.013 | 0.400 | 0.083 | 2.400 |
| **1** | 11.000 | 1600.000 | 3.100 | 830.000 | 670.000 | 250.000 | 3000.000 | 2600.000 | 0.180 | 0.580 | 0.330 | 18.000 | 0.340 | 0.017 | 0.670 | 0.000 | 0.740 |
| **2** | 4.000 | 1400.000 | 3.900 | 740.000 | 570.000 | 110.000 | 2200.000 | 3300.000 | 0.200 | 0.830 | 0.032 | 19.000 | 0.330 | 0.037 | 1.300 | 0.150 | 2.500 |
| **3** | 3.600 | 1900.000 | 4.600 | 820.000 | 860.000 | 80.000 | 2000.000 | 4000.000 | 0.190 | 1.000 | 0.150 | 26.000 | 0.240 | 0.036 | 1.500 | 0.042 | 3.000 |
| **4** | 4.800 | 1500.000 | 6.700 | 620.000 | 650.000 | 100.000 | 1600.000 | 3800.000 | 0.300 | 0.940 | 0.087 | 25.000 | 0.240 | 0.004 | 2.000 | 0.000 | 2.800 |
| **5** | 2.700 | 1500.000 | 5.200 | 550.000 | 670.000 | 20.000 | 2100.000 | 2300.000 | 0.077 | 1.000 | 0.480 | 23.000 | 0.210 | 0.042 | 1.400 | 0.036 | 2.000 |
| **6** | 5.900 | 1500.000 | 26.000 | 580.000 | 640.000 | 43.000 | 2600.000 | 2400.000 | 0.073 | 1.300 | 0.540 | 31.000 | 0.600 | 0.046 | 2.000 | 0.030 | 1.700 |
| **7** | 8.350 | 1900.000 | 6.000 | 615.000 | 855.000 | 48.000 | 3250.000 | 3000.000 | 0.135 | 1.900 | 0.325 | 33.500 | 0.461 | 0.024 | 2.250 | 0.115 | 2.610 |
| **8** | 12.000 | 3000.000 | 7.400 | 960.000 | 1300.000 | 61.000 | 6200.000 | 2600.000 | 0.110 | 2.700 | 0.670 | 55.000 | 0.510 | 0.032 | 1.700 | 0.680 | 4.800 |
| **9** | 34.000 | 5100.000 | 24.000 | 1000.000 | 2200.000 | 68.000 | 9900.000 | 3300.000 | 0.020 | 5.100 | 1.900 | 95.000 | 0.870 | 0.059 | 2.300 | 0.040 | 12.000 |
| **10** | 64.000 | 7100.000 | 29.000 | 890.000 | 3000.000 | 77.000 | 13000.000 | 3700.000 | 0.049 | 7.900 | 2.900 | 140.000 | 1.400 | 0.080 | 2.300 | 0.000 | 37.000 |
| **11** | 730.000 | 13000.000 | 1200.000 | 3500.000 | 1900.000 | 5300000.000 | 140000.000 | 2600000.000 | 41.000 | 18.000 | 69.000 | 30.000 | 4.400 | 15.000 | 580.000 | 94.000 | 46.000 |
| **12** | 190.000 | 4150.000 | 355.000 | 1500.000 | 530.000 | 1650000.000 | 18000.000 | 360000.000 | 11.000 | 7.750 | 30.000 | 8.400 | 1.300 | 2.500 | 210.000 | 24.000 | 18.500 |
| **13** | 74.107 | 1113.497 | 169.733 | 406.300 | 278.884 | 540214.432 | 19833.858 | 171185.369 | 7.287 | 3.116 | 23.170 | 5.411 | 0.632 | 0.709 | 165.729 | 10.364 | 8.555 |
| **14** | 33.087 | 724.930 | 96.484 | 126.872 | 230.228 | 204540.798 | 2863.589 | 97931.093 | 1.848 | 0.973 | 6.222 | 7.061 | 0.255 | 0.175 | 52.809 | 8.064 | 2.389 |
| **15** | 15.000 | 230.000 | 59.000 | 93.000 | 85.000 | 58000.000 | 1400.000 | 59000.000 | 1.400 | 0.490 | 1.300 | 6.200 | 0.190 | 0.056 | 20.000 | 6.400 | 1.600 |
| **16** | 16.750 | 302.500 | 57.250 | 82.250 | 72.250 | 54250.000 | 3050.000 | 55000.000 | 1.178 | 0.415 | 0.990 | 4.250 | 0.147 | 0.048 | 17.250 | 5.550 | 2.525 |
| **17** | 18.500 | 375.000 | 55.500 | 71.500 | 59.500 | 50500.000 | 4700.000 | 51000.000 | 0.955 | 0.340 | 0.680 | 2.300 | 0.105 | 0.041 | 14.500 | 4.700 | 3.450 |
| **18** | 16.151 | 311.096 | 41.975 | 63.253 | 46.873 | 41934.147 | 5857.624 | 45215.935 | 0.867 | 0.242 | 0.605 | 1.472 | 0.082 | 0.041 | 14.492 | 3.535 | 3.032 |
| **19** | 13.802 | 247.192 | 28.449 | 55.005 | 34.245 | 33368.294 | 7015.248 | 39431.871 | 0.779 | 0.144 | 0.530 | 0.645 | 0.059 | 0.041 | 14.483 | 2.370 | 2.614 |
| **20** | 9.093 | 193.693 | 17.671 | 44.522 | 22.776 | 25518.097 | 7444.936 | 32712.531 | 0.725 | 0.152 | 0.460 | 0.585 | -0.032 | 0.038 | 11.976 | 1.448 | 2.943 |
| **21** | 4.383 | 140.194 | 6.894 | 34.039 | 11.307 | 17667.899 | 7874.624 | 25993.191 | 0.671 | 0.160 | 0.391 | 0.525 | -0.123 | 0.034 | 9.468 | 0.527 | 3.272 |

| **Sample** | **Major elements** | | | | | | | | **Trace elements** | | | | | | | | |
| --- | --- | --- | --- | --- | --- | --- | --- | --- | --- | --- | --- | --- | --- | --- | --- | --- | --- |
|  | **Al** | **Ca** | **Fe** | **K** | **Mg** | **Na** | **S** | **Si** | **As** | **Co** | **Cu** | **Mn** | **Ni** | **Pb** | **Te** | **Ti** | **Zn** |
|  | **µg/L** | **µg/L** | **µg/L** | **µg/L** | **µg/L** | **µg/L** | **µg/L** | **µg/L** | **µg/L** | **µg/L** | **µg/L** | **µg/L** | **µg/L** | **µg/L** | **µg/L** | **µg/L** | **µg/L** |
| Detection limit | 1.000 | 1.000 | 0.250 | 1.000 | 0.500 | 10.000 | 25.000 | 50.000 | 0.025 | 0.025 | 0.025 | 0.010 | 0.100 | 0.010 | 0.010 | 0.050 | 0.100 |
| Blank H_2_O_2_ | 7.950 | 245.000 | 0.000 | 26.500 | 3.600 | 165.000 | 550.000 | 81.000 | 0.011 | 0.001 | 9.600 | 0.047 | 0.017 | 0.715 | 0.000 | 0.087 | 5.250 |
| Blank Si solution | 685 | 52 | 605 | 705 | 25 | 2600000 | 325 | 1900000 | 0.65 | 0.24 | 1.4 | 7.15 | 7.3 | 0.62 | 5.05 | 0.0002 | 0.000 |
